# Computational design of 6-substituted phenalenone fluorophores: Impact of electron-donating groups

**DOI:** 10.1007/s10895-026-04778-5

**Published:** 2026-04-24

**Authors:** Zelal Agin, Nuran Elmacı Irmak

**Affiliations:** Faculty of Science, Department of Chemistry, Izmir Institute of Technology, Urla, Izmir 35430 Turkey

**Keywords:** DFT, TD-DFT, Phenalenone, Fluorophore design, Fluorescent sensors, TICT

## Abstract

Fluorophores and their properties can be modified by substituents based on the application area. Phenalenone is commonly used as a photosensitizer due to its high singlet oxygen quantum yield. Also, its derivatives are used in sensor implementations. This study focused on designing novel phenalenone-based fluorophores by introducing electron-donating substituents at the 6-position and evaluating the influence of a spacer group on their photophysical properties through quantum chemical calculations. DFT (density functional theory) and TD-DFT (time-dependent density functional theory) calculations were performed on 6-substituted phenalenone-based fluorophores to investigate their photophysical properties. Moderate rotational changes in some derivatives supported ICT (intramolecular charge transfer), while others exhibited smaller geometric changes but still showed donor-to-acceptor charge transfer. Introducing an amino (–NH₂) spacer induced pronounced geometric changes around the C–N single bond, consistent with the TICT (twisted intramolecular charge transfer) mechanism and indicative of potential fluorescence quenching pathways with large Stokes shifts. In contrast, derivatives with minimal rotational changes and no charge transfer character exhibited smaller Stokes shifts and a potential for fluorescence, demonstrating promise as phenalenone-based fluorophores suitable for sensor applications. The findings of this study have provided fundamental insights for future experimental studies into fluorophore design for optical sensing systems.

**Supplementary Information:**

The online version contains supplementary material available at 10.1007/s10895-026-04778-5.

## Introduction

Fluorophores are molecules that emit light through the process of fluorescence. These can range from organic dyes and quantum dots to specific fluorescent proteins. The choice of fluorophore is typically guided by the specific application and the target analyte, ensuring optimal sensitivity and sensor performance.

Organic dyes are diverse and widely studied compounds that exhibit vivid colors due to their ability to absorb visible light. The optical properties of organic dyes, including their absorption spectra, fluorescence behavior, photo-thermal effects, and photodynamic capabilities, are crucial for determining their suitability for various applications. For instance, dyes that undergo color changes in response to external stimuli are advantageous for visual detection. On the other hand, near-infrared (NIR) fluorescent dyes are favored for biological imaging due to their minimal background interference. Moreover, photosensitizing dyes that generate reactive oxygen species (ROS) are widely used in cancer therapy [1]. Fluorophore dyes can be chemically modified to suit a wide range of applications. These modifications can improve properties such as fluorescence intensity, photostability, water solubility, and the ability to selectively target biological systems [2].

Phenalenone, also known as 1H-Phenalen-1-one, is a compound that consists of three fused phenyl rings with a ketone moiety. Certain derivatives of this compound are biosynthesized naturally in fungi and plants, including species within the Haemodoraceae and Musaceae families, as a response to microbial infection. Moreover, it serves as a highly efficient Type II photosensitizer known for its exceptional ability to generate singlet oxygen (^1^O_2_) with nearly 100% quantum yield [3]. Phenalenone plays an important role in photodynamic therapy (PDT) since this property. A group of researchers synthesized phenalenone derivatives with electron-donating and bromine groups. They suggested that moving the amino group from the 5- to 6-position caused a 100 nm red shift. One of their synthesized derivatives showed the longest absorption, strong fluorescence, efficient singlet oxygen production, and selective photocytotoxicity against pancreatic cancer cells [4].

In addition to its photosensitizing features, the use of phenalenone in sensor applications is noteworthy. In the design of these derivatives, photophysical properties, including absorption and emission wavelengths and intensities, as well as fluorescence quantum yields, are considered. Additionally, parameters such as selectivity, sensitivity, and photostability are considered based on the application area. For instance, Üçüncü studied a phenalenone derivative highly selective for Fe³⁺ ions. It was shown that the probe molecule remains unresponsive to other metal cations, confirming its high specificity for Fe^3+^ [5]. In another study, 3- and 6-dimethylamino phenalenone derivatives were synthesized by Sandoval-Altamirano et al. They revealed that the 3-substituted derivative illustrated weak emission due to close donor–acceptor proximity, while the 6-substituted one exhibited stronger fluorescence, solvatochromism, and a bathochromic shift in polar solvents, highlighting the effect of donor–acceptor distance on photophysical properties [6].

In this study, the ground- and excited-state properties of phenalenone-based organic dyes were investigated using quantum chemical methods. To this end, the ground- and excited-state geometries of phenalenone derivatives bearing different electron-donating groups at the 6-position of the phenalenone core were optimized through DFT and TD-DFT calculations. The 6-position was selected for donor substitution because it is commonly employed in the synthesis of various phenalenone derivatives. Such modifications are known to induce red shifts in the absorption spectra and to impart potential fluorescence sensing capabilities.

Within the scope of this work, eight phenalenone derivatives with different substituents at the 6-position were designed and evaluated for their fluorophore potentials. Their electronic structures and optical and photophysical characteristics were systematically analyzed. The influence of a spacer group (–NH₂) on both ground- and excited-state behavior was also assessed. Charge-transfer (CT) states were identified, and twisted intramolecular charge-transfer (TICT) states were examined in detail for derivatives exhibiting a twisted geometry in the excited state.

Twisted intramolecular charge transfer (TICT) refers to a charge-transfer process in molecules that contain electron donor and acceptor units connected by a π-conjugated spacer containing rotatable single bonds. A characteristic feature of TICT-forming dyes is the pronounced geometrical change that takes place in the excited state. Upon photoexcitation, a fluorescent molecule is promoted to an emissive excited state, which corresponds to a locally excited (LE) state in nonpolar systems or intramolecular charge transfer (ICT) in dipolar systems, and then returns to the ground state via radiative decay. In contrast, molecules capable of forming a TICT state initially populate the LE or ICT state after light absorption and subsequently undergo conformational relaxation. As the dihedral angle between the donor and acceptor moieties increases, the CT character of the excited state becomes progressively stronger. When this dihedral angle approaches 90°, a TICT state is established, characterized by near-complete electron transfer from the donor to the acceptor. In many organic dye systems, the TICT state predominantly relaxes to the ground state via non-radiative pathways, resulting in fluorescence quenching [7, 8].

## Computational Methods

According to the method validation calculations detailed in the Supporting Information (Tables S1 and S2), the B3LYP/def2-SVP level of theory with water as the solvent produced absorption wavelengths that most closely matched the experimental values reported in the literature for phenalenone derivatives. Therefore, this method was chosen for the computational analysis of the phenalenone derivatives. Additionally, D4 dispersion correction was included to improve accuracy. Therefore, all calculations were carried out at B3LYP/def2-SVP level of theory in water. The conductor-like polarizable continuum model (CPCM) was used for the solvation model. The Orca [9] and G16 [10] quantum chemistry packages were used to perform density functional theory (DFT) and time-dependent density functional theory (TD-DFT) calculations. The Conformer-Rotamer Ensemble Sampling Tool (CREST) program was used to determine low-energy conformers [11]. Since it uses semi-empirical tight-binding methods, the selected conformers were re-optimized with the DFT method. For the low-lying energy conformer of each derivative, vibrational frequency calculations were performed at the optimized geometries to confirm that the structures represent a minimum geometry. The charge distribution was analyzed using NBO calculations. TD-DFT calculations were performed to obtain excited-state structures, excitation energies, oscillator strengths, and dominant natural transition orbitals (NTOs). As a result of these calculations, the structural properties and dipole moments in the ground and excited states, as well as the electronic structures of the relevant molecules, were examined to reveal the CT character or the possibility of emission. The spin–orbit coupling (SOC) values, as well as the radiative and non-radiative decay rates, which include internal conversion and intersystem crossing, of the designed fluorophore molecules were computed. Furthermore, the theoretical fluorescence quantum yield was estimated as the ratio of the radiative rate to the total decay rate as mentioned in literature [12, 13]. On this basis, the fluorescence quantum yields of the investigated molecules were also evaluated via ORCA quantum chemistry package using the following Eq. (1):

1 $$\:FQY=\frac{{k}_{r,{S}_{1}{S}_{0}}}{{k}_{r,{S}_{1}{S}_{0}}+{k}_{ic,{S}_{1}{S}_{0}}+{k}_{isc,{S}_{1}{T}_{1}}}$$

Where $\:{k}_{r,{S}_{1}{S}_{0}}$is the radiative (fluorescence) rate of S_1_→S_0_, $\:{k}_{ic,{S}_{1}{S}_{0}}$is the internal conversion rate of S_1_→S_0_ and $\:{k}_{isc,{S}_{1}{T}_{1}}$is the intersystem crossing rate of S_1_→T_1_.

## Results and Discussion

**Fig. 1:**
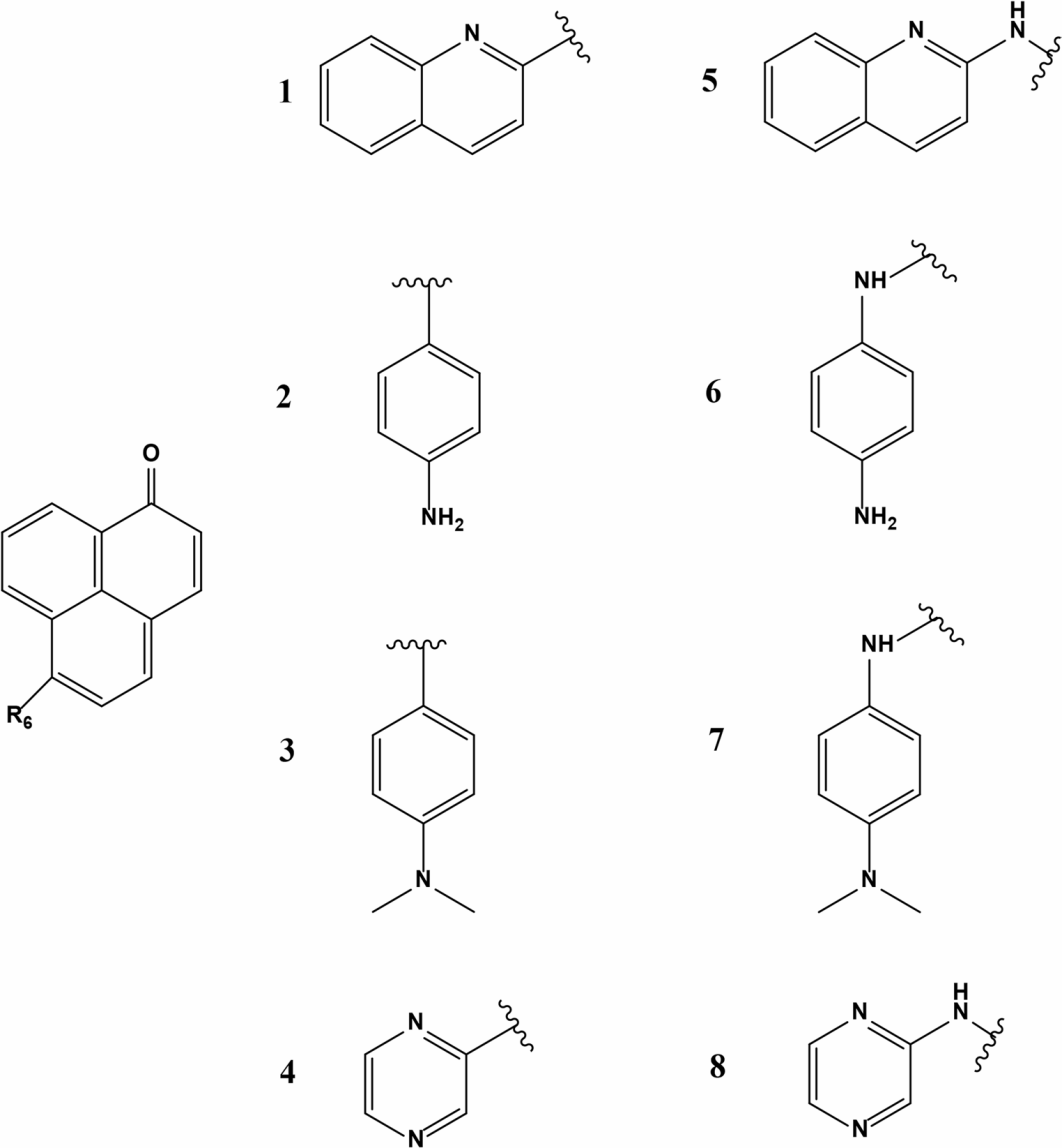
The substituents at the 6-position of the phenalenone molecule

The derivatives of phenalenone-based fluorophores with various electron-donating groups were attached at the 6-position of the phenalenone molecule. These donor moieties were given in Fig. 1.

**Fig. 2:**
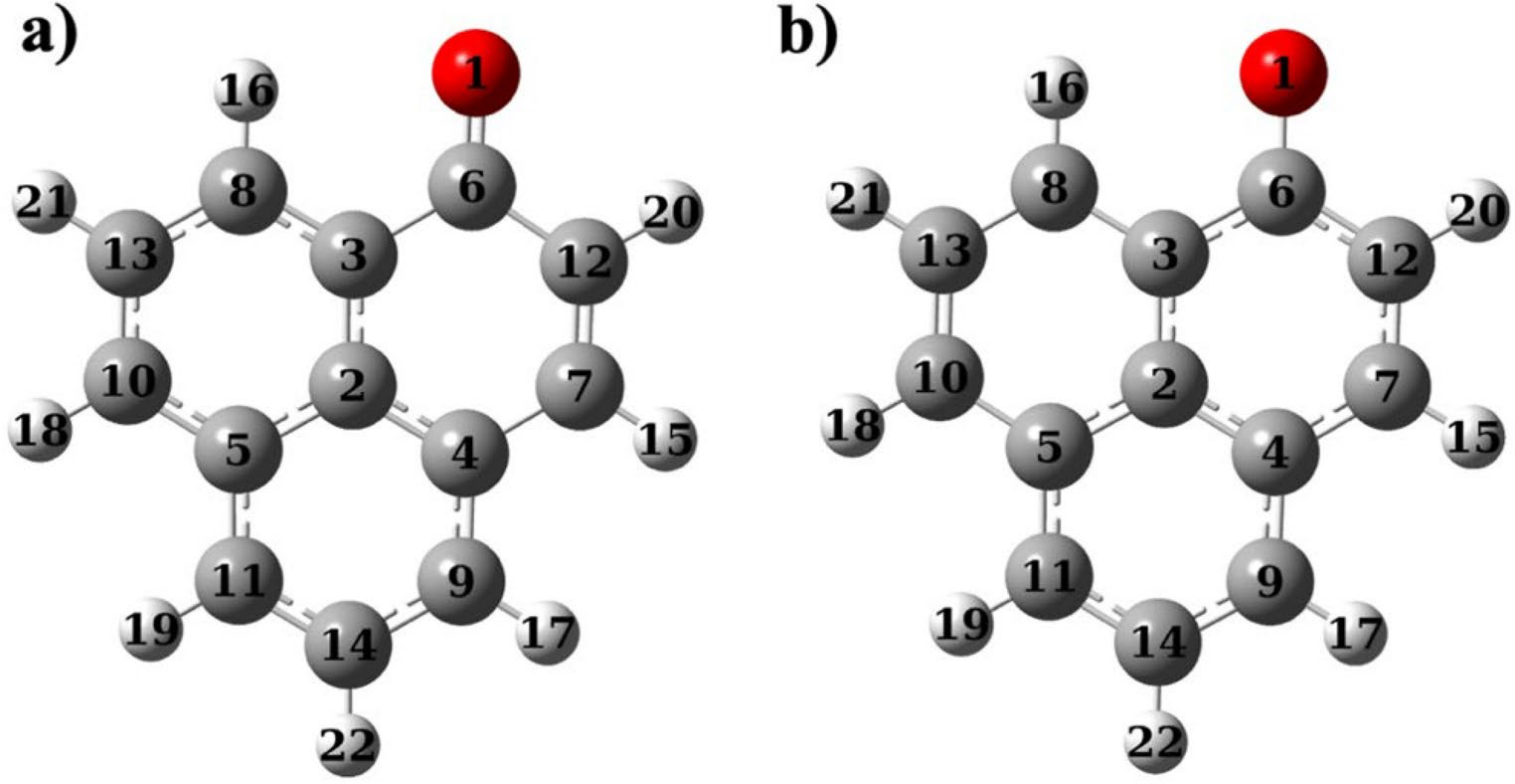
Optimized structures of unsubstituted phenalenone in ground state (**a**) and excited state (**b**)

The ground and excited state optimized geometries of unsubstituted phenalenone were shown in Fig. 2. Examination of the structural parameters revealed that the molecule undergoes minimal structural changes upon excitation, maintaining its overall planarity. In the excited state, the C = O double bond exhibited a shift toward single bond character, as indicated by an elongation of approximately 0.07 Å. This structural change promotes increased electron delocalization within the bi-phenyl rings connected to the carbonyl group. Additionally, the C = O bond became longer upon excitation in all molecules. Derivatives **1**, **2**, **3**, and **4** showed the same change in C = O bond length between the ground and excited states as their corresponding derivatives containing the –NH₂ group. Among the designed derivatives, molecule **3** exhibited the greatest bond length change, with an increase of 0.22 Å (Table 1).

**Table 1:** Dihedral angles and C = O bond distances of PN core and the designed derivatives in ground and excited states

Molecule	Dihedral angle (˚)				C = O bond distance (Å)		
	Dihedral atoms	Ground State	Excited State			Ground State	Excited State
unsubs. PN	O-C5-C11-C6	-180.0		-180.0	1.234		1.299
1	C5-C11-C22-N37	-44.6		-24.0	1.234		1.248
2	C5-C11-C22-C23	134.3		129.5	1.236		1.256
3	C5-C11-C22-C24	-135.6		-125.7	1.236		1.258
4	C5-C11-C22-N23	135.9		154.4	1.233		1.247
5	C5-C11-N22-C24	172.9		172.4	1.237		1.251
6	C5-C11-N22-C24	-170.6		-84.1	1.240		1.261
7	C5-C11-N22-C24	-170.9		-85.0	1.240		1.261
8	C5-C11-N22-C24	178.9		179.8	1.237		1.251

**Fig. 3:**
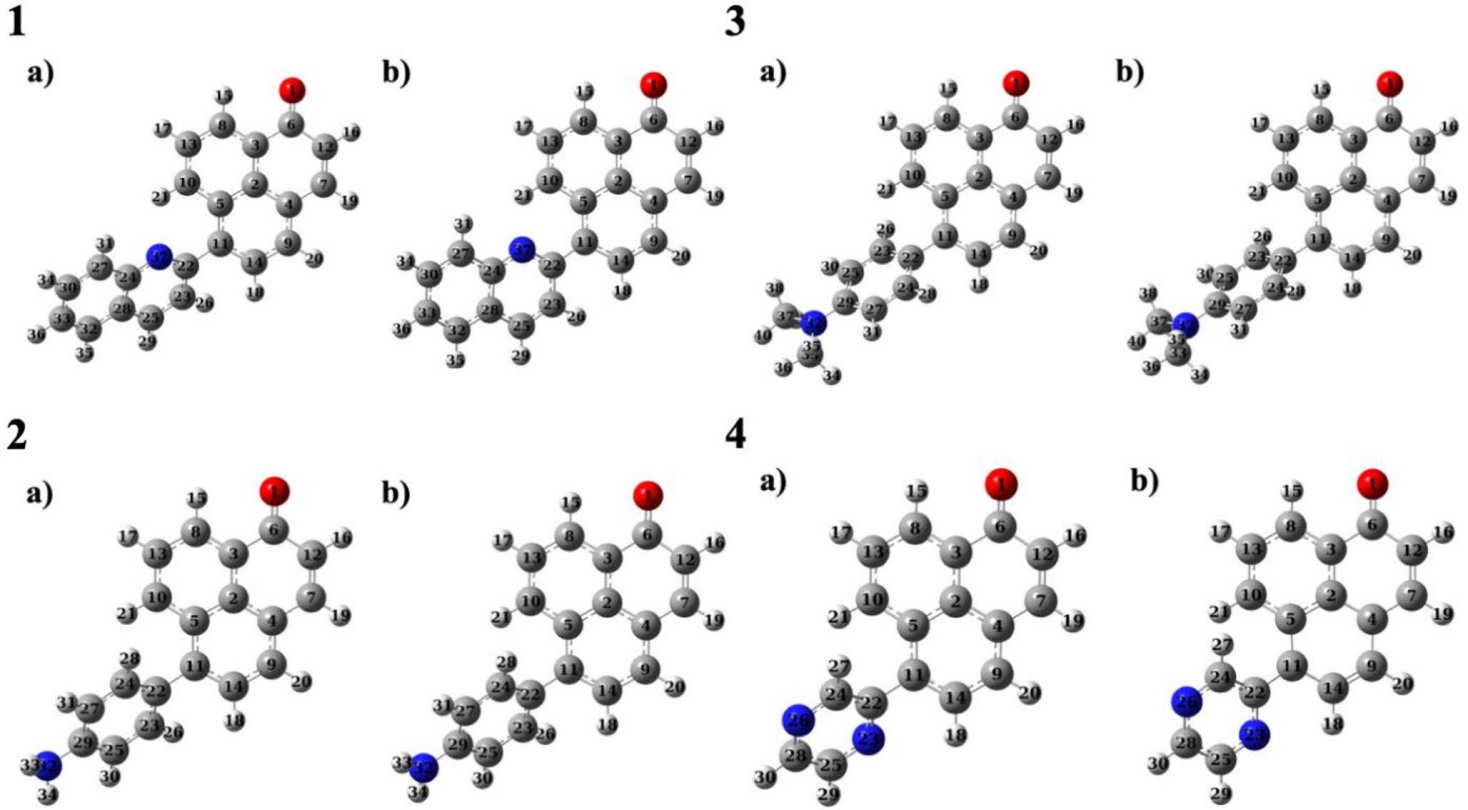
Optimized structures of the molecules 1–4 in ground state (**a**) and excited state (**b**)

The dihedral angles defined by C5-C11-C22-N37 atoms, at the ground and excited states, were obtained as -44.6˚ and − 24.0˚, respectively, in molecule **1**. This means there was a 20-degree rotation between the substituent and the phenalenone core in the anticlockwise direction, upon excitation. A similar situation was observed for molecule **4** with a rotation of 19 degrees (from 135.9˚ to 154.4˚, C5-C11-C22-N23). Notable rotational changes were observed in both derivatives, which may enable ICT. However, relatively less rotation was determined during the excitation in molecules **2** (C5-C11-C22-C23) and **3** (C5-C11-C22-C24) molecules compared to others, 5 and 10 degrees, respectively (Fig. 3).

**Fig. 4:**
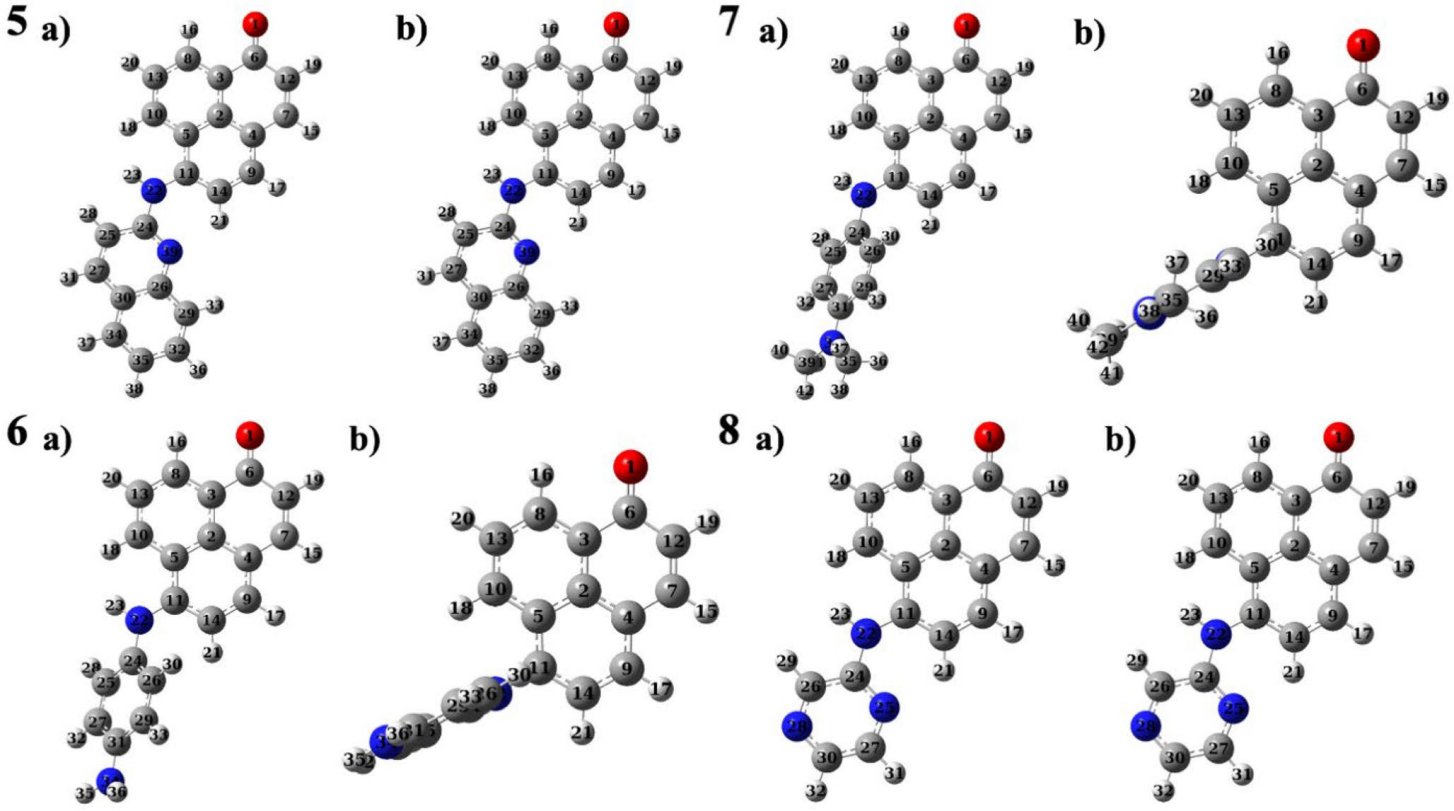
Optimized structures of the molecules 5–8 in ground (**a**) and excited (**b**) states

When an amino (-NH_2_) group was added between the substituent and phenalenone core as a spacer molecule, significant geometrical changes were detected in the corresponding derivatives. In contrast to molecules **1** and **4**, **5** and **8** were almost planar in both ground and excited states, that is, the substituent and PN core were in the same plane. The fact that the structures of these derivatives remained almost the same upon excitation decreased the potential exhibiting CT character and so supported the possibility of having fluorescence as a fluorophore. When donor and acceptor groups become perpendicular to each other upon excitation, molecules often show TICT behavior, characterized by high dipole moments, long emission wavelengths, large Stokes shifts, and typically quenched fluorescence [14]. The rotations, which were relatively less in molecules **2** and **3**, increased in their counterparts with -NH_2_ groups. The dihedral angles in the ground and excited states between C5-C11-N22-C24 were found as -170.6 and − 84.1 in molecule **6** and between C5-C11-N22-C24 were found as -170.9 and − 85.0 in molecule **7**, respectively. These rotational changes around the C-N single bond, approximately 85 degrees, supported the TICT process in both derivatives (Fig. 4).

**Fig. 5:**
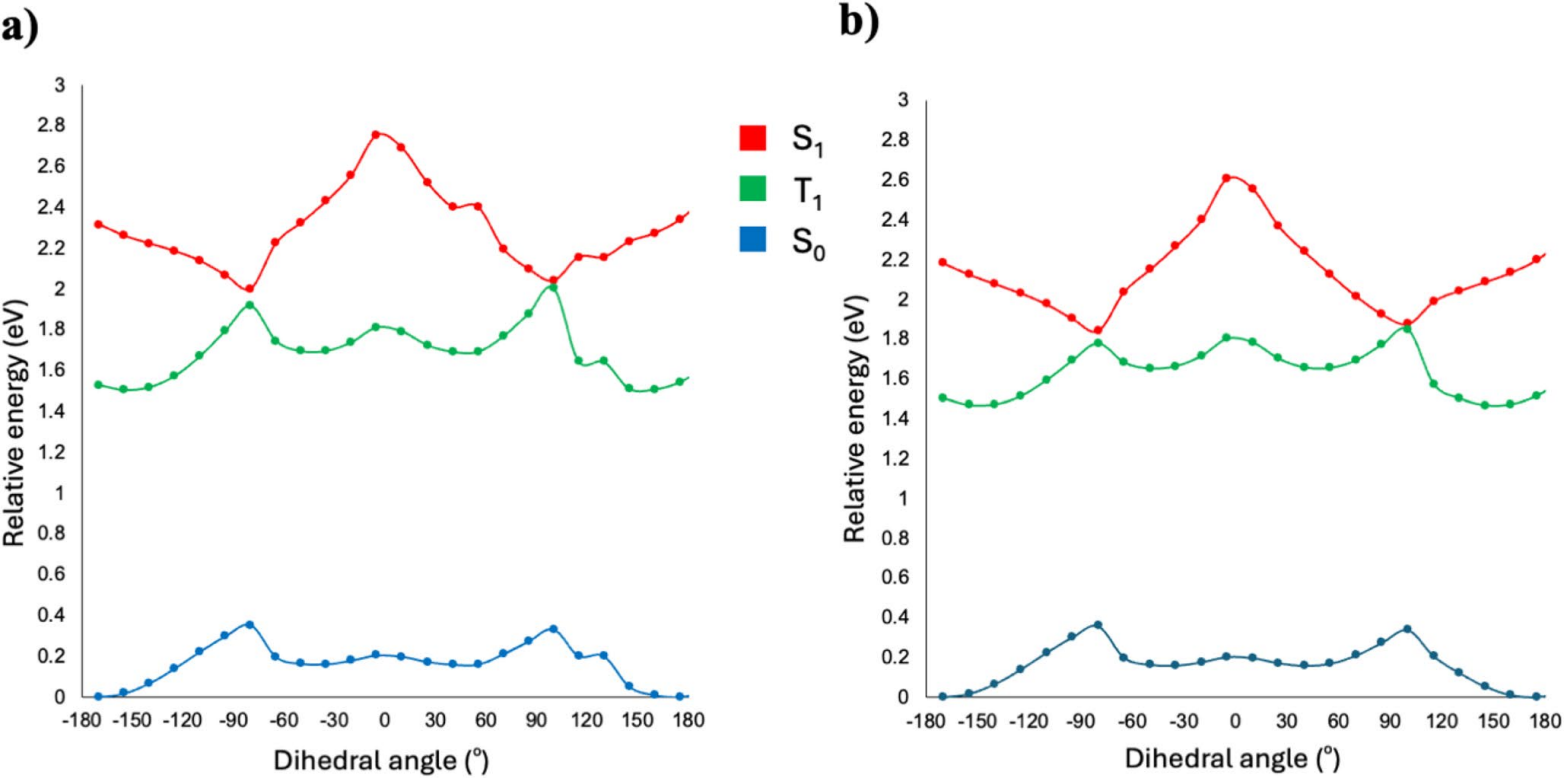
Potential energy surface sections of S_0_, S_1_ and T_1_ states with rotation angle between PN-substituent for derivative 6 (**a**) and 7 (**b**)

To further elucidate the TICT behavior of molecules **6** and **7**, potential energy surface (PES) profiles were constructed for the ground state (S₀) as well as the singlet and triplet excited states, based on the torsional angle between the donor and acceptor moieties. These molecules exhibited a planar configuration as the most stable geometry in the S₀ state, while a twisted structure became energetically favorable in the S₁ state, as illustrated in the potential energy surface section plots (Fig. 5). This observation suggested that excitation induced the formation of a TICT state for these molecules. Moreover, the overlap of the S₁ and T₁ states near 90° may enhance ISC efficiency, leading to non-radiative decay. In addition, the S₀ state reached a maximum whereas the S₁ state showed a minimum near a 90° dihedral angle, resulting in a red shift in emission spectra.

**Fig. 6:**
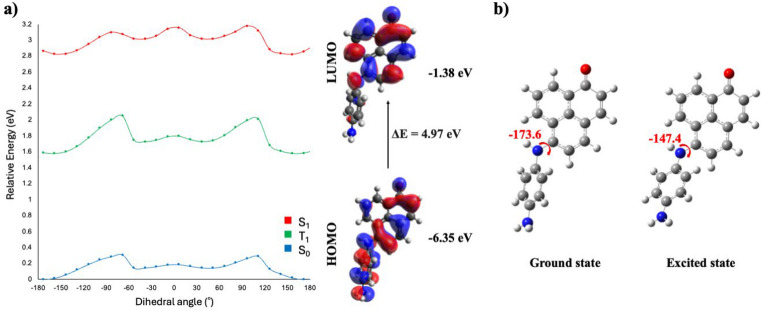
PES sections of S_0_, S_1_, T_1_ and HOMO-LUMO surfaces (**a**) and optimized structures (**b**) of molecule 6 obtained by CAM-B3LYP/def2-SVP calculations

**Fig. 7:**
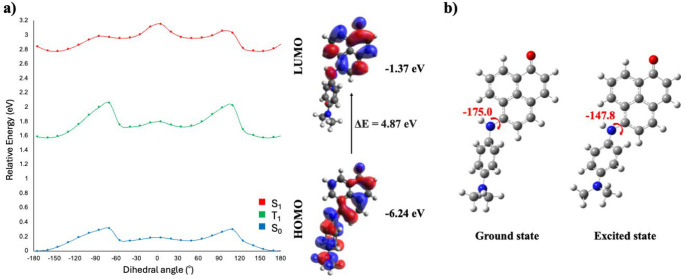
PES sections of S_0_, S_1_, T_1_ and HOMO-LUMO surfaces (**a**) and optimized structures (**b**) of molecule 7 obtained by CAM-B3LYP/def2-SVP calculations

Previous studies have reported that B3LYP tends to overstabilize charge transfer states [15]. In contrast, range-separated functionals such as CAM-B3LYP generally provide a more reliable description of CT states [16]. Therefore, to account for the possible overstabilization by B3LYP, molecules **6** and **7**, which displayed TICT behavior at the B3LYP level, were additionally investigated using the CAM-B3LYP functional. Although the CAM-B3LYP calculations did not predict a TICT state, they indicated the presence of an ICT from the phenyl unit of the substituent to one of the phenyl rings of the PN core in both molecules **6** and **7**, as evidenced by the FMOs shown in Figs. 6 and 7. In addition, a rotational change of approximately 27° upon excitation further supported the occurrence of the ICT process. These results indicated that the excited-state behavior of molecules **6** and **7** lies near the boundary between ICT and TICT regimes, where the qualitative description became strongly dependent on the choice of functional. Importantly, both methods consistently predicted the presence of charge transfer, differing mainly in the extent of twisting required for stabilization. In addition, it is important to note that the CPCM solvent model may underestimate solvation energies, which can result in inaccurate descriptions of CT states in aqueous solution. On the other hand, B3LYP has a tendency to overestimate the stability of CT states [17]. These opposing effects may partially compensate for one another, may yield reasonable results. Therefore, the observed differences between B3LYP and CAM-B3LYP reflected the sensitivity of the system to both the electronic structure method and solvation treatment.

The dipole moment values in both the ground and excited states offer insight into charge transfer behavior [18]. As shown in Table 2, the substitutions led to an increase in dipole moments compared to unsubstituted PN in both the S₀ and S₁ states (except molecule **4**). Additionally, for most derivatives, the dipole moments were higher in the excited state than in the ground state. Moreover, the significant difference in dipole moments between the S₀ and S₁ states for the derivatives **6** and **7** suggested the presence of charge separation, which facilitates the TICT process.

**Table 2:** Dipole moments (D) of PN core and the derivatives in ground and excited states

Molecule	unsubs. PN	1	2	3	4	5	6	7	8
Ground State	6.5766	9.2855	11.2483	11.8780	6.1594	10.6301	14.3931	15.0948	7.6785
Excited State	7.7570	10.1363	12.8337	12.5308	6.8817	11.6823	7.8299	7.7777	8.9126

**Fig. 8:**
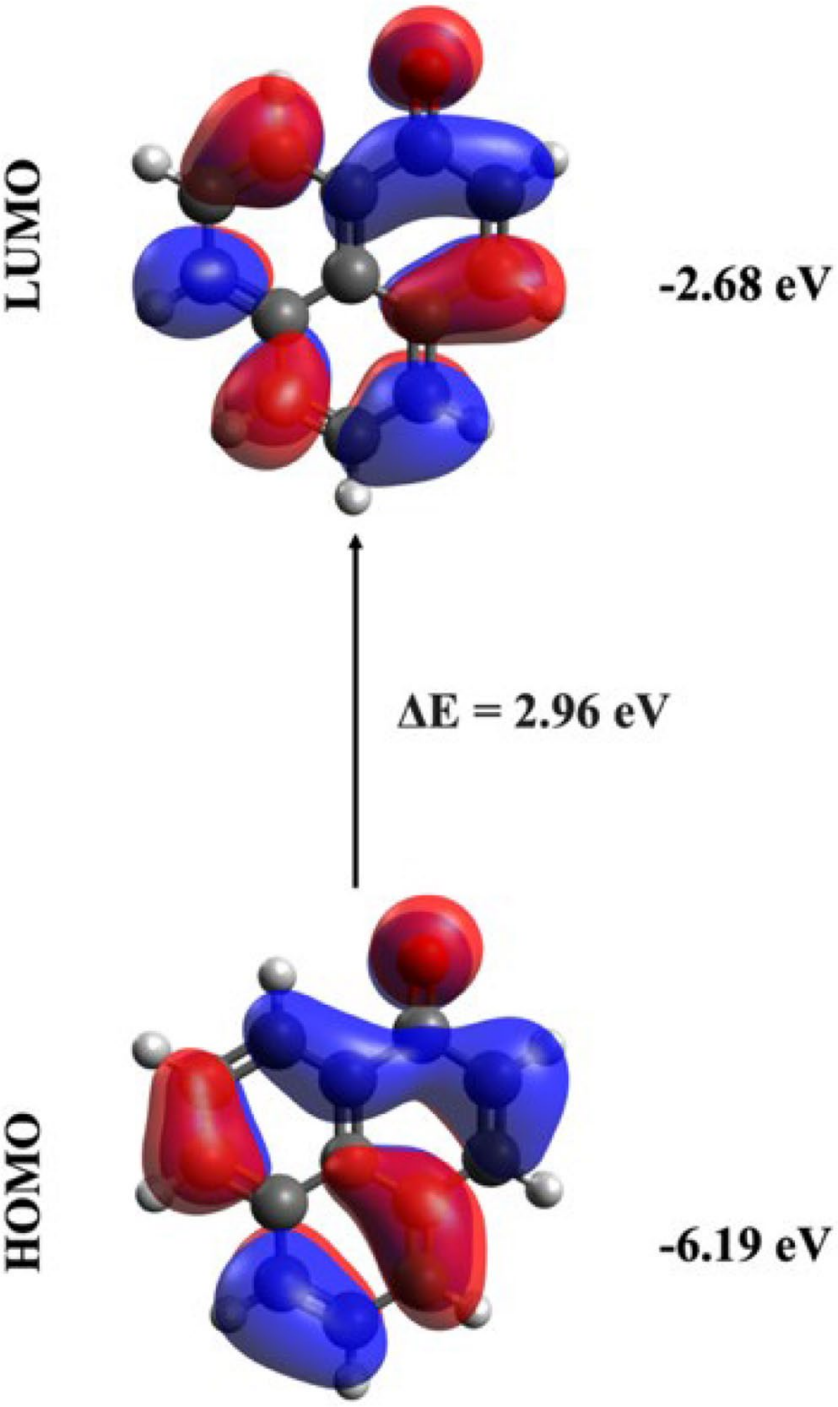
Frontier molecular orbitals and their energies of the unsubstituted phenalenone

In unsubstituted PN, S_1_ was forbidden state, whereas S_2_ state is allowed and corresponds to a H→L nature (π→π*), with 384 nm λ. Figure 8 showed that the electrons were delocalized over the entire molecule, with no evidence of charge separation.

Within the scope of this study, the absorption and emission maximum wavelengths of the designed phenalenone derivatives and their Stokes shifts were examined, and they were given in Table 3. Introducing electron donating groups to the 6-position caused a red shift in both absorption and emission spectra compared to the unsubstituted PN molecule. The largest shifts were observed in the derivative **7** with an absorption shift of about 190 nm and in derivatives **6** and **7** showing emission shifts around 1400 nm. It was observed that the emission wavelengths of **6** and **7** are in near-IR region (around 1800 nm) while their absorption fell into VIS region (around 550 nm), indicating large Stokes shifts. These pronounced shifts, along with notable structural changes upon excitation, suggested TICT behavior. It should be noted that the absorption and emission wavelengths of compounds **6** and **7** calculated at the CAM-B3LYP level lied in the visible region, and unlike the B3LYP results, they did not exhibit TICT behavior but they possess ICT character.

**Table 3:** Absorption, emission wavelengths and Stokes shifts of the phenalenone derivatives

Derivative	λabs (nm)	λems (nm)	Stokes shift
unsubs. PN	384	484	100
1	415	520	105
2	525	633	108
3	572	664	92
4	408	510	102
5	481	556	75
6	543/437*	1886/600*	1343/163*
7	578/440*	1780/600*	1202/160*
8	468	546	78

*The wavelengths were obtained by CAM-B3LYP/def2-SVP in water level of theory

In contrast, derivatives **2** and **3** exhibited smaller geometric changes upon excitation, resulting in reduced Stokes shifts and thus greater environmental stability. Additionally, derivatives **5** and **8** showed smaller Stokes shifts, whereas their non-NH_2_ counterparts undergo structural rotations that led to longer emission wavelengths and, consequently, larger Stokes shifts.

**Fig. 9:**
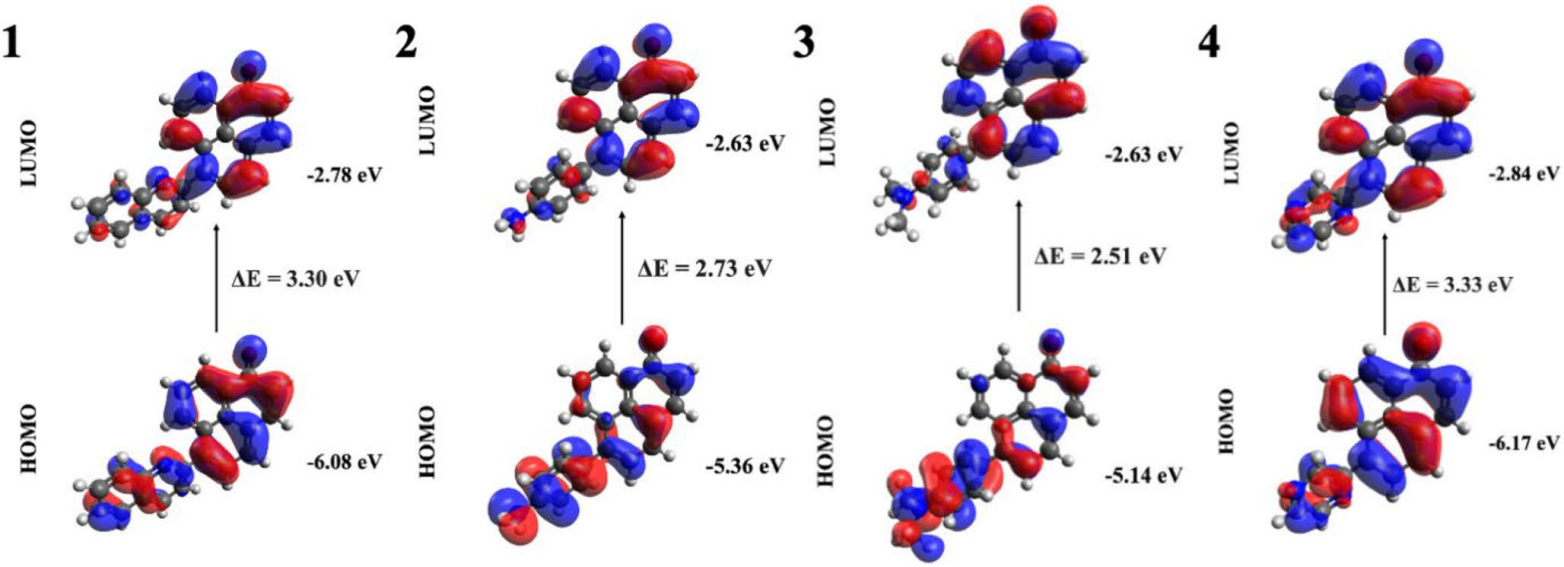
Frontier molecular orbitals and excitation energies of the molecules 1–4

H→L transition had the highest contribution to the most intense absorption for all derivatives (Table 4). The S₁ state was a bright state due to its high oscillator strength in derivatives **1–3**, while S_2_ state was the allowed one for molecule **4**. In compound **1**, it exhibited a maximum absorption (λₘₐₓ) at 415 nm, associated with π→π* character. Analysis of the Frontier molecular orbitals (FMOs) showed partial electron migration from the donor substituent to the acceptor PN core. In molecule **2**, a strong absorption is observed at 525 nm (λₘₐₓ). The S₁ state in this compound represented a charge transfer transition from the donor to the PN core, indicating ICT behavior. For molecule **3**, absorption λₘₐₓ appeared at 572 nm, signifying charge transfer from the donor to the acceptor. Additionally, the rotation between the substituent and the PN core upon excitation suggested a possible pathway for fluorescence quenching. The introduction of methyl groups to the nitrogen atom of the substituent raises the HOMO energy, reducing the energy gap and causing a 47 nm red shift in absorption. In molecule **4**, the most intense transition was S₀→S₂, observed at 408 nm λₘₐₓ, characterized by a π→π*. The electrons were delocalized over the conjugated π-system. Since no charge transfer was evident from the FMOs, **4** was expected to exhibit fluorescence, indicating potential for emission (Fig. 9).

**Table 4:** Vertical transitions, oscillator strengths and MO contributions of the PN derivatives

Molecule	Transition	ƒ	Nature of transition	Contribution	Molecule	Transition	ƒ	Nature of transition	Contribution
unsubs. PN	S0→S1	0.0000	H-1 → L	0.974	5	S0→S1	0.6419	H → L	0.973
			H-1 → L + 3	0.021		S0→S2	0.0000	H-3 → L	0.926
	S0→S2	0.2183	H-3 → L	0.011				H-3 → L + 1	0.011
			H-2 → L	0.043				H-2 → L	0.035
			H → L	0.921		S0→S3	0.0161	H-1 → L	0.776
1	S0→S1	0.4031	H-4 → L	0.011				H → L + 1	0.199
			H-2 → L	0.217	6	S0→S1	0.4927	H → L	0.985
			H → L	0.739		S0→S2	0.0294	H-4 → L	0.012
	S0→S2	0.1188	H-3 → L	0.025				H-2 → L	0.039
			H-2 → L	0.715				H-1 → L	0.923
			H-2 → L + 1	0.011		S0→S3	0.0017	H-2 → L	0.934
			H → L	0.219				H-2 → L + 4	0.015
	S0→S3	0.0262	H-1 → L	0.965				H-1 → L	0.038
			H → L + 1	0.011	7	S0→S1	0.4884	H → L	0.991
2	S0→S1	0.3600	H → L	0.986		S0→S2	0.0793	H-4 → L	0.011
	S0→S2	0.0014	H-2 → L	0.965				H-1 → L	0.959
	S0→S3	0.1125	H-4 → L	0.041		S0→S3	0.0003	H-2 → L	0.971
			H-1 → L	0.921				H-2 → L + 4	0.016
3	S0→S1	0.3725	H → L	0.992	8	S0→S1	0.5504	H → L	0.970
	S0→S2	0.0014	H-2 → L	0.966		S0→S2	0.0000	H-1 → L	0.958
	S0→S3	0.1583	H-4 → L	0.032				H-1 → L + 5	0.011
			H-1 → L	0.932		S0→S3	0.0392	H-4 → L	0.013
4	S0→S1	0.0000	H-2 → L	0.182				H-2 → L	0.026
			H-1 → L	0.778				H → L + 1	0.941
	S0→S2	0.4368	H-3 → L	0.017					
			H → L	0.955					
	S0→S3	0.0081	H-2 → L	0.749					
			H-2 → L + 1	0.038					
			H-1 → L	0.177					
			H-1 → L + 1	0.015					

**Fig. 10:**
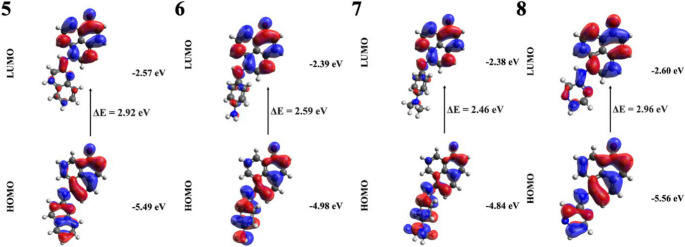
Frontier molecular orbitals and excitation energies of the molecules 5–8

In molecule **5**, the electronic transition from the ground state to the first excited state occurred at 481 nm with partial CT character from the donor moiety (2-amino quinoline) to the acceptor moiety (PN). Analysis of the molecular geometries in both the ground and excited states suggested the potential for fluorescence emission, as complete charge transfer was not achieved. Additionally, in molecule **6**, state 1 had a λ_max_ of 543 nm. Evaluation of HOMO and LUMO electron density distributions revealed a charge transfer from the 4-aminoaniline donor to the phenalenone acceptor. Additionally, the rotation of the substituent upon excitation suggested the involvement of a TICT mechanism. As a result, fluorescence quenching was expected in this derivative. In the case of compound **7**, transition to S_1_ had λ_max_ at 578 nm with a significant CT character, thereby enhancing the possibility of TICT mechanism; hence, fluorescence quenching of this derivative was anticipated. Conversely, for **8**, the S₁ absorption was strong at λₘₐₓ = 468 nm and corresponded to a π→π* transition without net charge transfer (Fig. 10). Given the similar optimized geometry of this derivative in both the ground and excited states, the probability of emission (fluorescence) from this derivative was comparatively high.

When the compounds **1**, **2**, **3** and **4** were compared with their counterparts having -NH_2_ group, the addition of electron donating spacer group to the derivatives caused an increase in the energy of both HOMO orbitals and LUMO orbitals of the four molecules mentioned, thus decreasing the energy gap, which led to a noticeable red shift in the absorption wavelength. Furthermore, when the oscillator strengths were examined, the addition of the spacer group caused an increase in intensity in all derivatives.

The radiative (k_fluor_) and non-radiative rates including intersystem crossing (k_isc_) and internal conversion (k_ic_) of the relevant molecules, were calculated to analyze how excited molecules relax after absorbing light (see Table 5). From these results, the theoretical fluorescence quantum yields (FQYs) were also determined. Among the designed molecules, **4**, **5**, and **8** exhibited high fluorescence rates, whereas molecules **2** and **8** showed comparatively low intersystem crossing rates. Nevertheless, all derivatives displayed very low fluorescence quantum yields. This behavior can be attributed to the dominance of internal conversion in all compounds, with k_ic_ consistently exceeding both k_fluor_ and k_isc_. Although molecule **4** possessed the largest k_isc_ value and a relatively SOC constant (see Table S3), its excited-state deactivation was still primarily governed by internal conversion. Furthermore, all derivatives exhibited large S₁–T₁ energy gaps (Table S5) and generally small SOC values (Table S3, S4), which were consistent with inefficient intersystem crossing. Despite variations in SOC strengths and singlet–triplet energy separations across the series, ISC remained weak due to the substantial S₁–T₁ gaps (0.66–1.18 eV relative to the ground state). Overall, these results suggest that non-radiative decay pathways, particularly internal conversion, play a dominant role in deactivating the excited states, leading to weak fluorescence efficiency in all compounds. It should be noted that the present rate calculations are based on a limited number of electronic states (S₀, S₁, and T₁) and therefore are intended to provide qualitative trends rather than quantitative predictions of fluorescence quantum yields.

**Table 5:** Calculated fluorescence (k_fluor_), intersystem crossing (k_isc_), internal conversion (k_ic_) rates and fluorescence quantum yields (FQY) of the designed phenalenone derivatives

Molecule	k_fluor_ (s^− 1^)	k_isc_ (s^− 1^)	k_ic_ (s^− 1^)	FQY
1	2.17 × 10^8^	5.45 × 10^5^	1.40 × 10^10^	0.015
2	1.42 × 10^8^	5.75 × 10^1^	8.62 × 10^10^	0.002
3	7.06 × 10^7^	1.25 × 10^4^	4.05 × 10^9^	0.017
4	2.65 × 10^8^	7.68 × 10^6^	3.68 × 10^10^	0.007
5	3.19 × 10^8^	1.58 × 10^5^	2.00 × 10^11^	0.002
6	7.38 × 10^5^	3.85 × 10^4^	1.70 × 10^7^	0.041
7	1.02 × 10^6^	6.09 × 10^5^	3.10 × 10^8^	0.003
8	3.01 × 10^8^	4.53 × 10^3^	1.44 × 10^11^	0.002

## Conclusions

In this study, DFT and TD-DFT calculations were carried out on phenalenone derivatives with electron-donating moieties at the 6-position to investigate their photophysical properties. Moderate rotational changes in the ground and excited state geometries were observed in derivatives **1** and **4**, which may facilitate the ICT character. Although molecules **2** and **3** exhibited relatively smaller rotations upon excitation, HOMO to LUMO surfaces showed a charge transfer from donor to acceptor moieties. The introduction of an amino (-NH₂) group as a spacer induced pronounced geometric changes in the corresponding derivatives. Unlike **1** and **4**, the **5** and **8** derivatives remained nearly planar in both ground and excited states, supporting their potential as fluorophores. Furthermore, the limited rotations seen in **2** and **3** increased in their -NH₂-substituted counterparts (molecules **6** and **7**). At the B3LYP level, the PES sections of molecules **6** and **7** exhibited a minimum at a twisted geometry, consistent with a TICT state. In contrast, the CAM-B3LYP PES sections did not show a stable minimum at full twisting but instead rotated by 27° at S_1_ state supported an ICT state with partial rotation. Notably, both methods consistently predicted the presence of charge transfer, differing mainly in the extent of geometrical relaxation. In contrast, derivatives **2**, **3**, **5**, and **8** underwent smaller geometric changes during excitation, leading to reduced Stokes shifts and enhanced environmental stability. Moreover, the electrons were delocalized across the conjugated π-system, and the absence of charge transfer in the FMOs suggested that it is likely to exhibit fluorescence in molecule **4** with a pyrazine moiety. Derivatives **5** and **8**, which contain 2-amino quinolone and 2-amino pyrazine, respectively, had the potential for fluorescence emission, as complete charge transfer was not achieved, as indicated by molecular orbital plots. Based on the rate constant calculations, compounds **4**, **5**, and **8** demonstrated high fluorescence rate constants, while compounds **2** and **8** exhibited relatively low intersystem crossing rates. However, despite these favorable rates, all of the derivatives were characterized by very low fluorescence quantum yields due to dominant internal conversion rates.

Overall, derivative **8** had almost no structural deviation and does not display net charge transfer upon excitation. It produced emission at 546 nm in the visible range and featured a high radiative decay rate. Therefore, it represents a promising candidate as a phenalenone-based fluorophore. This research provided a valuable basis for future experimental investigations into fluorophore design for sensing technologies.

## Supplementary Information

Below is the link to the electronic supplementary material.

Supplementary Material 1 (DOCX 83.0 KB)

## Data Availability

The datasets generated during and/or analysed during the current study are available from the corresponding author on reasonable request.
