# Supplementary material for: Computational design of 6-substituted phenalenone fluorophores: Impact of electron-donating groups
Source: J Fluoresc. 2026 Apr 24;36(5):3353–63. doi: 10.1007/s10895-026-04778-5 (PMC13226320; doi:10.1007/s10895-026-04778-5)
Supplement: Supplementary file 1 — Supplementary Material 1 (DOCX 83.0 KB) [file 10895_2026_4778_MOESM1_ESM.docx]

***Supporting Information***

**Computational Design of 6-Substituted Phenalenone Fluorophores: Impact of Electron-Donating Groups**

Zelal Agin and Nuran Elmacı Irmak^*^

Faculty of Science, Department of Chemistry, Izmir Institute of Technology, 35430, Urla, Izmir, Turkey

Corresponding author e-mail: nuranelmaci@iyte.edu.tr

Geometry optimizations and frequency calculations were carried out in acetonitrile using the basis sets and functionals specified in Table S1, and the resulting optimized structures were subsequently employed in TD-DFT calculations to obtain the absorption wavelengths. The calculated results were compared against available experimental absorption data in the literature for 3-hydroxyphenalenone (3-OHPN, 349 nm), 6-hydroxyphenalenone (6-OHPN, 425 nm), 3-ethoxyphenalenone (3-OEtPN, 324 nm), 6-ethoxyphenalenone (6-OEtPN, 427 nm), 3-dimethylaminophenalenone (3-DMAPNF, 343 nm), and 6-dimethylaminophenalenone (6-DMAPNF, 471 nm) in order to select the most appropriate basis set and functional for the quantum chemical calculations [1],[2]. Since some reference molecules exhibit experimental absorption spectra in PBS solution (pH ≈ 7.4), geometry optimizations, frequency, and TD-DFT calculations were performed in both the gas phase and water to make a more accurate decision on the selection of the suitable method (Table S2). The results were compared with literature data for 6-aminophenalenone (6-AP, 548 nm), 5-aminophenalenone (5-AP, 457 nm), 9-methoxyphenalenone (9-OMetPN, 418 nm), 2-bromo-9-methoxyphenalenone (9-OMet-2BrPN, 427 nm), and 6-bromo-5-aminophenalenone (5-NH₂-6BrPN, 458 nm) [3]. The average error was calculated based on the experimental absorption wavelength values, and B3LYP/def2-SVP yielded the lowest error of 42.1. As a result, it was found appropriate to calculate the phenalenone derivatives determined for this thesis study with the B3LYP/def2-SVP method in water. Since DFT methods do not include dispersion forces, D4 dispersion correction was added to this method to enhance accuracy.

**Table S1** Calculated absorption wavelengths of phenalenone derivatives with different functionals and basis sets in acetonitrile

|  |  | **def2-SVP** | | | | | | | | **6-31G(d)** | |
| --- | --- | --- | --- | --- | --- | --- | --- | --- | --- | --- | --- |
| **Molecule** | **Solvent** | **B3LYP** | **error** | **CAM-B3LYP** | **error** | **PBE0** | **error** | **WB97X-D4** | **error** | **B3LYP** | **error** |
| **3-OHPN** | ACN | 384.1 | -35.1 | 348.6 | 0.4 | 374.0 | -25.0 | 333.6 | 15.4 | 376.2 | - |
| **6-OHPN** | ACN | 418.8 | 6.2 | 380.7 | 44.3 | 408.5 | 16.5 | 363.0 | 62.0 | 417.0 | - |
| **3-OEtPN** | ACN | 375.4 | -51.4 | 333.1 | -9.1 | 363.2 | -39.2 | 316.5 | 7.5 | 373.5 | -49.5 |
| **6-OEtPN** | ACN | 417.8 | 9.2 | 373.5 | 53.5 | 406.0 | 21.0 | 353.9 | 73.1 | 414.8 | 12.2 |
| **3-DMAPNF** | ACN | 432.7 | -89.7 | 359.8 | -16.8 | 417.4 | -74.4 | 333.1 | 9.9 | 421.9 | -78.9 |
| **6-DMAPNF** | ACN | 470.5 | 0.5 | - | - | 458.7 | 12.3 | 381.9 | 89.1 | 460.8 | 10.2 |

**Table S2** Calculated absorption values of phenalenone derivatives with different functionals and basis sets in water and gas phase

| **Basis Set** | **Functional** | **6-AP** | **5-AP** | **9-OMetPN** | **9-OMet-2BrPN** | **5-NH2-6BrPN** |
| --- | --- | --- | --- | --- | --- | --- |
| **def2-SVP** | **B3LYP** | 423.1 | 444.2 | 377.2 | 391.8 | 451.2 |
|  | **error** | 124.9 | 12.8 | 40.8 | 35.2 | 6.8 |
|  | **B3LYP (in water)** | 458.0 | 495.1 | 386.2 | 409.7 | 491.2 |
|  | **error** | 90.0 | -38.1 | 31.8 | 17.3 | -33.2 |
|  | **CAM-B3LYP** | 374.8 | 380.3 | 339.4 | 347.0 | 383.6 |
|  | **error** | 173.2 | 76.7 | 78.6 | 80.0 | 74.4 |
|  | **CAM-B3LYP (in water)** | 413.9 | 412.4 | 349.2 | 358.5 | 409.4 |
|  | **error** | 134.1 | 44.6 | 68.8 | 68.5 | 48.6 |
|  | **PBE0** | 411.4 | 428.4 | 366.5 | 377.7 | 434.6 |
|  | **error** | 136.6 | 28.6 | 51.5 | 49.3 | 23.4 |
|  | **PBE0 (in water)** | 445.9 | 476.2 | 375.2 | 395.2 | 473.1 |
|  | **error** | 102.1 | -19.2 | 42.8 | 31.8 | -15.1 |
|  | **WB97X-D4** | 351.8 | 352.8 | 324.1 | 327.8 | 355.4 |
|  | **error** | 196.2 | 104.2 | 93.9 | 99.2 | 102.6 |
|  | **WB97X-D4 (in water)** | 393.7 | 377.0 | 333.7 | 339.2 | 373.8 |
|  | **error** | 154.3 | 80.0 | 84.3 | 87.8 | 84.2 |
| **6-31G(d)** | **B3LYP** | 415.4 | 433.7 | 374.8 | - | - |
|  | **error** | 132.6 | 23.3 | 43.2 | - | - |
|  | **B3LYP (in water)** | 448.1 | 474.6 | 384.2 | - | - |
|  | **error** | 99.9 | -17.6 | 33.8 | - | - |

**Table S3** Calculated spin-orbit coupling (SOC) values of molecules 1-4

| **Root** | | **Molecule 1** | **Molecule 2** | **Molecule 3** | **Molecule 4** |
| --- | --- | --- | --- | --- | --- |
| **Triplet** | **Singlet** | **SOC** | **SOC** | **SOC** | **SOC** |
| 1 | 0 | 0.244 | 0.380 | 0.405 | 0.484 |
| 1 | 1 | 23.302 | 0.352 | 0.289 | 23.258 |
| 1 | 2 | 0.405 | 19.454 | 18.692 | 1.350 |
| 1 | 3 | 0.490 | 0.637 | 0.712 | 3.224 |
| 1 | 4 | 3.325 | 0.525 | 0.489 | 0.805 |
| 1 | 5 | 1.100 | 0.234 | 0.214 | 0.474 |
| 2 | 0 | 35.271 | 3.189 | 1.935 | 35.579 |
| 2 | 1 | 0.350 | 1.660 | 1.097 | 1.445 |
| 2 | 2 | 16.423 | 16.378 | 16.714 | 18.447 |
| 2 | 3 | 6.373 | 2.217 | 1.714 | 0.239 |
| 2 | 4 | 2.852 | 1.221 | 0.871 | 5.087 |
| 2 | 5 | 2.803 | 0.163 | 0.255 | 5.174 |
| 3 | 0 | 2.367 | 34.587 | 34.778 | 0.724 |
| 3 | 1 | 7.415 | 10.904 | 9.693 | 3.255 |
| 3 | 2 | 1.045 | 2.600 | 2.010 | 0.681 |
| 3 | 3 | 0.148 | 16.415 | 16.874 | 0.566 |
| 3 | 4 | 5.855 | 7.341 | 6.405 | 0.212 |
| 3 | 5 | 1.940 | 1.381 | 1.197 | 0.266 |
| 4 | 0 | 5.050 | 0.206 | 0.260 | 3.166 |
| 4 | 1 | 1.037 | 0.190 | 0.202 | 1.228 |
| 4 | 2 | 1.830 | 1.131 | 1.471 | 2.701 |
| 4 | 3 | 2.020 | 0.328 | 0.318 | 0.205 |
| 4 | 4 | 2.048 | 0.166 | 0.164 | 0.555 |
| 4 | 5 | 0.928 | 0.233 | 0.229 | 0.909 |
| 5 | 0 | 5.265 | 0.578 | 0.521 | 0.410 |
| 5 | 1 | 1.860 | 0.302 | 0.261 | 2.856 |
| 5 | 2 | 1.625 | 0.794 | 0.923 | 0.996 |
| 5 | 3 | 1.781 | 0.214 | 0.227 | 0.343 |
| 5 | 4 | 0.224 | 0.104 | 0.083 | 0.228 |
| 5 | 5 | 0.241 | 0.139 | 0.147 | 0.313 |

**Table S4** Calculated spin-orbit coupling (SOC) values of molecules 5-8

| **Root** | | **Molecule 5** | **Molecule 6** | **Molecule 7** | **Molecule 8** |
| --- | --- | --- | --- | --- | --- |
| **Triplet** | **Singlet** | **SOC** | **SOC** | **SOC** | **SOC** |
| 1 | 0 | 0.323 | 1.131 | 1.111 | 0.010 |
| 1 | 1 | 0.248 | 0.228 | 0.221 | 0.036 |
| 1 | 2 | 22.329 | 12.920 | 12.333 | 22.749 |
| 1 | 3 | 0.115 | 1.026 | 1.470 | 0.030 |
| 1 | 4 | 0.122 | 0.483 | 0.493 | 2.309 |
| 1 | 5 | 0.137 | 0.079 | 0.102 | 0.017 |
| 2 | 0 | 35.071 | 0.616 | 0.523 | 35.432 |
| 2 | 1 | 15.462 | 0.512 | 0.488 | 16.306 |
| 2 | 2 | 0.085 | 21.222 | 21.156 | 0.071 |
| 2 | 3 | 6.202 | 1.647 | 2.607 | 3.552 |
| 2 | 4 | 2.369 | 0.064 | 0.071 | 0.961 |
| 2 | 5 | 7.872 | 0.157 | 0.136 | 4.012 |
| 3 | 0 | 0.530 | 34.903 | 34.976 | 0.037 |
| 3 | 1 | 0.077 | 7.519 | 6.964 | 0.022 |
| 3 | 2 | 12.919 | 1.318 | 2.042 | 14.605 |
| 3 | 3 | 0.083 | 16.666 | 16.044 | 0.020 |
| 3 | 4 | 0.231 | 0.332 | 0.220 | 2.141 |
| 3 | 5 | 0.116 | 0.822 | 0.476 | 0.010 |
| 4 | 0 | 0.595 | 0.945 | 0.898 | 0.054 |
| 4 | 1 | 0.163 | 0.583 | 0.603 | 0.017 |
| 4 | 2 | 3.777 | 0.699 | 1.125 | 4.131 |
| 4 | 3 | 0.229 | 0.070 | 0.114 | 0.017 |
| 4 | 4 | 0.095 | 0.273 | 0.258 | 1.020 |
| 4 | 5 | 0.149 | 0.211 | 0.220 | 0.000 |
| 5 | 0 | 0.782 | 0.148 | 0.333 | 0.041 |
| 5 | 1 | 0.147 | 0.522 | 0.478 | 0.033 |
| 5 | 2 | 4.051 | 2.923 | 3.861 | 5.997 |
| 5 | 3 | 0.060 | 0.199 | 0.467 | 0.000 |
| 5 | 4 | 0.036 | 0.105 | 0.108 | 0.522 |
| 5 | 5 | 0.051 | 0.171 | 0.175 | 0.010 |

**Table S5** Singlet and triplet state excitation energies of molecules 1-8 (in eV)

| State/Molecule | 1 | 2 | 3 | 4 | 5 | 6 | 7 | 8 |
| --- | --- | --- | --- | --- | --- | --- | --- | --- |
| S1 | 2.635 | 2.305 | 2.203 | 2.614 | 2.543 | 1.788 | 1.704 | 2.629 |
| S2 | 2.762 | 2.821 | 2.824 | 2.827 | 2.830 | 2.889 | 2.880 | 2.808 |
| S3 | 3.036 | 3.264 | 3.186 | 3.119 | 3.314 | 3.044 | 2.993 | 3.591 |
| S4 | 3.222 | 3.648 | 3.637 | 3.426 | 3.534 | 3.307 | 3.281 | 3.614 |
| S5 | 3.426 | 3.787 | 3.760 | 3.733 | 3.604 | 3.691 | 3.663 | 3.689 |
| T1 | 1.458 | 1.376 | 1.362 | 1.431 | 1.486 | 1.057 | 1.042 | 1.500 |
| T2 | 2.336 | 2.337 | 2.233 | 2.314 | 2.538 | 2.030 | 1.988 | 2.511 |
| T3 | 2.654 | 2.535 | 2.535 | 2.811 | 2.764 | 2.598 | 2.588 | 2.911 |
| T4 | 2.779 | 3.028 | 3.026 | 2.907 | 3.073 | 2.996 | 2.999 | 3.119 |
| T5 | 2.999 | 3.205 | 3.198 | 3.010 | 3.152 | 3.097 | 3.082 | 3.165 |

Cartesian coordinates of molecule **1** in ground state

O 5.62404568155822 -1.66638678008148 0.07408213233828

C 2.41810430495602 0.10103037657494 -0.00267269405380

C 3.34545328260750 -0.96905991802915 0.16255242571996

C 2.89141343757790 1.40363567121000 -0.35164515754799

C 1.01879701225660 -0.12982626003345 0.19265072085109

C 4.80442011043634 -0.75251059421403 -0.04887262291249

C 4.31167605215910 1.61065359571856 -0.54980654776390

C 2.88881762997671 -2.22941785230871 0.53027618790028

C 1.97885946057258 2.44359356045208 -0.48490242809377

C 0.60400059157475 -1.43097663974527 0.58655537143485

C 0.10143369791920 0.96696336529276 0.01928928576324

C 5.21416395105096 0.60763444125510 -0.41166242845664

C 1.51968818693514 -2.45663577234679 0.75101446624054

C 0.60281578761392 2.22460809696164 -0.29607581921722

H 3.61907301168311 -3.03222179118064 0.65241276870286

H 6.28377582969661 0.77173645318676 -0.56363091772285

H 1.17485386235388 -3.44741729138897 1.05653689785671

H -0.08670263126182 3.05960200819723 -0.43487120642418

H 4.64212365757403 2.61857481460151 -0.81840184386884

H 2.33519029781804 3.44236667201925 -0.75023388861793

H -0.45416660636716 -1.62086488311536 0.75443226750015

C -1.37283100291800 0.80622599747711 0.15175813921573

C -2.12588369271713 1.79168250871542 0.86103935626662

C -3.29192469852762 -0.38948196517436 -0.35193503906221

C -3.49121678682050 1.65790632458020 0.95017153198419

H -1.61395587616049 2.62732948984273 1.34031765022571

C -3.89308668512839 -1.51587212288755 -0.98004891777494

C -4.12906302872798 0.54920773868504 0.33495642796387

H -4.09210105481893 2.39147488183669 1.49476428366405

C -5.25929991026230 -1.69890868825119 -0.92511510258713

H -3.24124508788851 -2.22062772691894 -1.50121357173328

C -5.53258370127631 0.33397000771072 0.37553155918595

C -6.08659754875911 -0.76846864258591 -0.24285644384347

H -5.71368921752429 -2.56690997100199 -1.40999487764668

H -6.16197036454911 1.05565316645190 0.90340760430948

H -7.16694366966439 -0.93051094459608 -0.21043975584539

N -1.94234428294857 -0.23905132690984 -0.42556981395088

Cartesian coordinates of molecule **1** in excited state

O 5.59489772141810 -1.72044584265319 -0.11855118310813

C 2.41380761345360 0.08392131348918 -0.00089785937184

C 3.33212484557515 -0.99604637138824 0.04653387204498

C 2.90887194645347 1.42422764983069 -0.21133790952646

C 1.00598722238927 -0.15599281587950 0.17555133110427

C 4.78034930819094 -0.77546416884643 -0.14868796076142

C 4.29847471083834 1.64184815166235 -0.39276231012483

C 2.87170981438447 -2.30736690273132 0.29314639476117

C 1.98374818918977 2.51064285028125 -0.21640234268949

C 0.59681718240458 -1.48492868484943 0.45124041432322

C 0.09191577561419 0.96723628431371 0.11025587051502

C 5.20122090989007 0.59736890392711 -0.37113339228428

C 1.51797335888162 -2.53664712190573 0.50419813244912

C 0.64241356527239 2.28800640242319 -0.04474962801349

H 3.60495345447431 -3.11472689923058 0.32257227389033

H 6.27088860145245 0.76994244422502 -0.51702365247901

H 1.15945080982227 -3.54772724701619 0.71501340188215

H -0.03339375323565 3.14069880200489 -0.09243661454439

H 4.64625595878381 2.66643691396291 -0.55121200713977

H 2.35958667613713 3.52429053282532 -0.37273191548681

H -0.45634097621721 -1.68681909232032 0.61981686286488

C -1.35598772211799 0.84685012538725 0.14903582755551

C -2.16707812369489 1.95853818306306 0.58313415437042

C -3.26617856870991 -0.44079327708383 -0.21083321938160

C -3.52956498867649 1.83487503542733 0.63023395078778

H -1.70005839801213 2.88154580490191 0.92302526183339

C -3.84400774299583 -1.67859848582587 -0.64166529101155

C -4.14500629084768 0.61723265759103 0.22365964729712

H -4.15341651550758 2.65970534841068 0.98429170912154

C -5.21015763685699 -1.85157949899745 -0.63014292335161

H -3.16781463057662 -2.46953578463781 -0.97301369991380

C -5.54045862327178 0.40837527148778 0.22207854079046

C -6.06674581450808 -0.80506375984724 -0.19574051242431

H -5.64481461029587 -2.79930253141817 -0.95774205160812

H -6.19830142256877 1.21674700689202 0.55197815154953

H -7.14844098249563 -0.96019993582975 -0.19437500020828

N -1.93058086403682 -0.30855126164568 -0.23252632371174

Cartesian coordinates of molecule **2** in ground state

O 5.17358433067017 -1.27103853518885 -0.02839582003077

C 1.78779693020153 0.12748945252588 0.00222922329574

C 2.82751245852767 -0.84703610954452 0.05261079042463

C 2.11626059882779 1.51290951756942 -0.10778355800554

C 0.41829098402543 -0.28394600075164 0.08421173239178

C 4.25792473878452 -0.44163204233590 -0.04851751839925

C 3.50599519674534 1.90206196946048 -0.19258030073256

C 2.50863595779304 -2.19179633448150 0.21173695714845

C 1.08743231445659 2.45165470517803 -0.11859499940227

C 0.14892624664583 -1.66327890865053 0.28618109299659

C -0.62561088832308 0.71073212729390 0.00895860971633

C 4.51632172265768 0.99386524098044 -0.16875855181533

C 1.17088614004608 -2.59791223741291 0.34452916527741

C -0.25763247431349 2.05382730732550 -0.06341465005435

H 3.32339500838917 -2.91801742137347 0.25053071962249

H 5.56358713894473 1.29888162957997 -0.23573323031712

H 0.93398641947542 -3.65252771858645 0.50409416823236

H -1.03796107447975 2.81541031440081 -0.12097334619994

H 3.72793707766439 2.97027361267393 -0.27773075258974

H 1.33307370843014 3.51443372070643 -0.19538692651439

H -0.88171744885259 -1.99264281988794 0.41533658443038

C -2.06030040607518 0.35509521528757 0.00145909013494

C -2.97534729806201 1.05225034155667 0.81633557977842

C -2.58515029161376 -0.63320481809652 -0.85715275041806

C -4.33529388711570 0.76474355896435 0.79969664511172

H -2.60650999882919 1.82525519988985 1.49555229899773

C -3.94498374223125 -0.92092353272419 -0.89244685089567

H -1.91811042033676 -1.16531658551930 -1.53921291425383

C -4.85460089617066 -0.23457882977255 -0.05536303298692

H -5.01720358741718 1.31128277800924 1.45718893222068

H -4.32330036444675 -1.68121287846697 -1.58146508433803

N -6.19080326861400 -0.55599669659760 -0.04254278217925

H -6.55856714573972 -1.06408611427534 -0.84003055677218

H -6.83385377966447 0.12728089226372 0.34373203612550

Cartesian coordinates of molecule **2** in excited state

O 5.16784296326006 -1.29458824425768 0.07175472896090

C 1.78184536460870 0.12871602887893 0.00561823196095

C 2.82324378520082 -0.84251051868255 0.09778583049195

C 2.10822428822232 1.52266764208487 -0.14354618609048

C 0.40629172039300 -0.28456787636585 0.07583296813631

C 4.23964792369396 -0.44989456443811 0.01337892558725

C 3.48717288059523 1.90049481161267 -0.21173383663487

C 2.49358056970441 -2.20374791502113 0.27293228856937

C 1.06763199581138 2.47452916254754 -0.20276741198690

C 0.12716596849703 -1.66880208333480 0.26721300221102

C -0.61738184717033 0.71594772172750 -0.00833416717774

C 4.49524113047149 0.96906561471436 -0.14234822425025

C 1.16014218604095 -2.60120946248160 0.36295959321351

C -0.26269514310686 2.07241454967576 -0.12181203388718

H 3.30981465457917 -2.92494391830797 0.33768083930122

H 5.54502780868687 1.27252458474621 -0.19997285690776

H 0.91514319952485 -3.65725547310823 0.51004139722381

H -1.05458281285266 2.82386460885395 -0.17564504021544

H 3.72443611474036 2.96293215753068 -0.32304239301340

H 1.31566957471946 3.53404062027921 -0.30504774045971

H -0.90520922064134 -2.00615740359406 0.35779499221572

C -2.05441047457317 0.37318001296380 -0.01726779361501

C -2.95577312160144 1.02052660455766 0.87017994965067

C -2.58901344119834 -0.56227937923179 -0.94461428287492

C -4.30678849224590 0.74940690627070 0.85124412380328

H -2.56336264966369 1.74237008006109 1.58911605329219

C -3.93472897455167 -0.85272045521053 -0.98011230341712

H -1.91849912246820 -1.05185685936672 -1.65254950287035

C -4.83232541369967 -0.20346554076001 -0.07538455177465

H -4.99036090303084 1.24922663921144 1.54107368531646

H -4.33507729485214 -1.57129818233423 -1.69857164357813

N -6.14101212419283 -0.48153998548395 -0.09850044625002

H -6.52426839809161 -1.15653553013454 -0.75399536181629

H -6.78803269480935 -0.02623435360262 0.53893916688557

Cartesian coordinates of molecule **3** in ground state

O 5.84053024677008 -1.39948211783997 0.03433628014217

C 2.50462809691674 0.11530638354880 0.00861313227663

C 3.51105921177293 -0.89298845076538 -0.05522021806764

C 2.87967695735558 1.48604450305981 0.14860107694199

C 1.12229525963216 -0.24714843770524 -0.09061190782646

C 4.95371512828306 -0.53930455485765 0.06401828887136

C 4.28082476418271 1.82537783133700 0.24932626448662

C 3.14768042172056 -2.22240729233075 -0.24546820910805

C 1.88299243510934 2.45920208043809 0.17212605421842

C 0.80828048591125 -1.61133302821040 -0.32702074143845

C 0.11163667849914 0.78074916647031 0.00160339065488

C 5.25997982763562 0.88360592215684 0.21396784349168

C 1.79826211353681 -2.57909822754579 -0.39834280307472

C 0.52601050321887 2.10922003575687 0.10256916512564

H 3.93727738500969 -2.97534485027582 -0.29360245793913

H 6.31646305931594 1.15156466317747 0.29357366731396

H 1.52685081143485 -3.62105463042018 -0.58409895605487

H -0.22760882360866 2.89612033263131 0.17337084168479

H 4.53861832165308 2.88356982504667 0.35643770160574

H 2.16466871977645 3.51115880691115 0.27157298827869

H -0.23146518377785 -1.90244180867425 -0.47347796760723

C -1.33290395445153 0.47540792996226 -0.00168517626449

C -1.89583627690561 -0.52813437699080 0.81280815868965

C -2.22720109214573 1.23602450758490 -0.78112167073623

C -3.26334278540895 -0.77331202730324 0.84009751946555

H -1.25071688074199 -1.11263547206502 1.47266768804400

C -3.59600853165657 0.99837082677803 -0.77764825262335

H -1.83480994617885 2.02405497294045 -1.42920233251149

C -4.16074929083734 -0.02380253859126 0.03242894433439

H -3.63906458129832 -1.54948683114361 1.50547977924925

H -4.23263787705632 1.60904365401377 -1.41656932262187

N -5.50961336187188 -0.27223437923418 0.03828840373014

C -6.40534137484168 0.55050732326830 -0.75415608223081

H -6.37872526435643 1.61209376896961 -0.44539949197803

H -6.15512219149225 0.50986601698877 -1.82919028154353

H -7.43292185154196 0.18622935379010 -0.63330740417203

C -6.05647610984370 -1.29956263740463 0.90570228587294

H -5.62426866965289 -2.29219693385072 0.68705975270085

H -5.87285226492442 -1.08410605162308 1.97479014428048

H -7.14078411514189 -1.36204325799855 0.75268390433855

Cartesian coordinates of molecule **3** in excited state

O 5.82892837743964 -1.41600470036697 -0.15710395207422

C 2.49444230139908 0.12323058407129 -0.00146662997405

C 3.50116079002508 -0.87778611937332 -0.15354433886312

C 2.86837666309611 1.49210427199721 0.23299895410813

C 1.10456499607190 -0.23720305518252 -0.09308544599078

C 4.93109930602008 -0.54179338037168 -0.04756799135054

C 4.25944858224693 1.81458534804103 0.32047424987527

C 3.12210298938651 -2.21309588023728 -0.40844291532238

C 1.85990785809494 2.47226639936174 0.35749334521550

C 0.77584451604670 -1.59669757471915 -0.36266536296053

C 0.11657912812556 0.79002289973442 0.05745865400909

C 5.23492104750202 0.85366533998724 0.19214489700071

C 1.77517144819873 -2.55767213117745 -0.51669157670988

C 0.51541246355778 2.12115593256810 0.25998959964237

H 3.91182033398659 -2.95792047757394 -0.51906786137061

H 6.29453021453360 1.11704137809988 0.26617076711669

H 1.49318380948330 -3.59431869673304 -0.72387899096544

H -0.25131268221165 2.89331787113383 0.36510090866354

H 4.53514335994896 2.85899224607456 0.49642132177977

H 2.14401157030447 3.51430437499978 0.52576393834764

H -0.26902533165106 -1.89092829366792 -0.46319685965520

C -1.33283242919924 0.48847738572918 0.03823878282344

C -1.90874517274302 -0.43421943697106 0.94561628688404

C -2.20087558241444 1.15900091806793 -0.85645282749945

C -3.26444751198525 -0.68918806221077 0.95810273251357

H -1.26572069256916 -0.95296953137717 1.65863296337672

C -3.56238897350970 0.92824434007296 -0.85854343705874

H -1.78144219580203 1.87567818579738 -1.56531228034323

C -4.13803547164356 -0.01246193813763 0.05025724898291

H -3.66090491196437 -1.40835007087012 1.67205022181094

H -4.18999792230216 1.47060671121966 -1.56291318796059

N -5.47411162664175 -0.25583098956058 0.05400438851108

C -6.35947200878997 0.42690466745093 -0.88367701862140

H -6.33191859510396 1.51604026808121 -0.72090352186933

H -6.05859789644460 0.22015903066995 -1.92236440888622

H -7.38322596377344 0.07106440829243 -0.73265946863771

C -6.05781297618609 -1.20246579838349 0.99868992730474

H -5.63989506133925 -2.21018194030833 0.84729993950996

H -5.85528111255159 -0.89165752875692 2.03535597190696

H -7.14033655164166 -1.23994509287136 0.84377880253036

Cartesian coordinates of molecule **4** in ground state

O 4.82279008601165 1.09891948587052 -0.01151368899522

C 1.37823685202156 -0.13955140330626 -0.01512838572835

C 2.45993741162117 0.78635063700062 -0.07685748609135

C 1.64313351915115 -1.53854493420184 0.10328898500995

C 0.02929105989394 0.33363539876664 -0.09197150611961

C 3.87107658255295 0.31519500100724 0.01933537679407

C 3.01721675186568 -1.99208776315919 0.18539201142489

C 2.20232832108076 2.14257317167817 -0.23870643692885

C 0.57855241413864 -2.43352311457091 0.12259987530578

C -0.18075984365548 1.72512837099704 -0.29135208721342

C -1.04392287171700 -0.62024869649472 -0.01352415097259

C 4.06550266397152 -1.13224593029789 0.15018371915042

C 0.88212346479800 2.60981532949014 -0.36002355842262

C -0.74904016238718 -1.97586982758057 0.06080007527689

H 3.04847591072615 2.83133946880341 -0.28576586587872

H 5.09851983257387 -1.48265592252485 0.21435001299500

H 0.69247073306497 3.67412947838362 -0.51709738159370

H -1.57067091063069 -2.69329421471653 0.09799643229667

H 3.18871511772212 -3.06868299595280 0.27751648503954

H 0.77799643789708 -3.50543979995808 0.20048201944978

H -1.19396272871082 2.10822552506246 -0.41330054496793

C -2.47361450657369 -0.22082277918142 0.00665659451310

N -3.32251717695906 -0.91913463245132 -0.76409635080781

C -2.95898545314837 0.79213092408336 0.85838366771800

C -4.61279890133057 -0.59002217404657 -0.71506907414661

N -4.24962305256203 1.12082143425310 0.90466806677920

H -2.28007015060512 1.32497480978538 1.53132128725900

C -5.07909117773423 0.44028773838319 0.10926552250071

H -5.30678260890444 -1.15658504280609 -1.34575026024010

H -6.14172761417255 0.70518245768413 0.12751664659387

Cartesian coordinates of molecule **4** in excited state

O 4.80534811396923 1.10610441805201 0.20977892052026

C 1.37758932374188 -0.12310953272978 -0.03532107084849

C 2.45720595645836 0.78876408333071 -0.00012487313161

C 1.63175488542890 -1.54586174265864 0.04979447487578

C 0.03248745085749 0.36334733815695 -0.17993454858944

C 3.84445987270453 0.31221775913838 0.16714321250999

C 2.96158773580517 -2.00737665190111 0.19952313976665

C 2.22083158615781 2.17685647597344 -0.14652259943094

C 0.53457301151181 -2.45776788286570 -0.06010696220914

C -0.15087089718358 1.75403678866928 -0.39546608727331

C -1.04634157298096 -0.58473413721419 -0.14615716730158

C 4.02666539861800 -1.12706859777500 0.26881724990554

C 0.92996114039210 2.64235333460356 -0.36271783959220

C -0.74851497530679 -1.99233925024274 -0.17622123738153

H 3.07737400662356 2.85158142732948 -0.11247064423553

H 5.05050278670328 -1.48812243493335 0.39648624854624

H 0.74985984905169 3.70850988720463 -0.52178232144616

H -1.58900560286828 -2.68321880095415 -0.24266971084755

H 3.13592655244873 -3.08520824088452 0.25648233837877

H 0.73714899208968 -3.53101177950924 -0.03843071441664

H -1.14093569949373 2.14308529087204 -0.62871706706975

C -2.44892599690026 -0.21915037181188 -0.03325619213039

N -3.36792544371113 -1.08823790607867 -0.52807849172001

C -2.89620603262478 0.93553926635773 0.66922662280735

C -4.64790517972787 -0.76934876784511 -0.40959205437169

N -4.18124595256417 1.24863009255937 0.77926228616535

H -2.18164843307089 1.57386598736632 1.19566814002464

C -5.06617250522395 0.41915926023515 0.21352912481861

H -5.38730815440855 -1.46554942691369 -0.82189431108263

H -6.12747021649730 0.68405411446870 0.26335213475941

Cartesian coordinates of molecule **5** in ground state

O -6.50898555256663 0.07504835765437 -0.12616191468277

C -2.84747276461765 0.12831668495855 0.01161442790484

C -4.17135917657677 -0.39445688851999 -0.07319359627541

C -2.64720680862878 1.53947391153730 0.09667340355941

C -1.72905315172963 -0.76453360756131 0.00253937365842

C -5.35467000000024 0.51450742128503 -0.06062063744944

C -3.79309520880940 2.41275233821642 0.10189733510381

C -4.37155509745721 -1.76764391843347 -0.17422733193781

C -1.34256736372908 2.02579750991248 0.16682736045819

C -1.98501555487811 -2.15178045026135 -0.12031888412040

C -0.38899181304771 -0.21127090494952 0.10537502170893

C -5.06894130288352 1.94289107810465 0.03068176677146

C -3.27899883814792 -2.64600996853911 -0.20390439157786

C -0.22974348912412 1.17731640560910 0.17100474462779

H -3.60818317228434 3.48962511783012 0.16634123054801

H -5.39587819149341 -2.14075697036837 -0.23700365074126

H -1.18173605413845 3.10596311824427 0.22793455379435

H -1.16729667664821 -2.87379482507045 -0.16533668801902

H -5.92883073661878 2.61731282196336 0.03736276957866

H -3.44199207708808 -3.72204724996003 -0.29777747143777

H 0.77498426958283 1.58315662135272 0.23009285329785

N 0.67064603135316 -1.09328602318741 0.14557944333908

H 0.42138814195146 -2.07168153052484 0.21920461952120

C 2.04390405121642 -0.89047986335537 0.11091675692616

C 2.85608883273051 -2.07149501230997 0.20634223271352

C 3.90617279014608 0.46750710114179 -0.05170593474806

C 4.21716751455656 -1.93878921480653 0.17061115783742

H 2.38145932873262 -3.05059218674284 0.30362346009334

C 4.45297109421706 1.77164120734661 -0.18647189112618

C 4.80149658698256 -0.64537012093602 0.03791444181208

H 4.86464142695096 -2.81696555698666 0.24190943497577

C 5.82228763472305 1.95751337134980 -0.22936463856602

H 3.76417545074798 2.61677589965203 -0.25590654026599

C 6.20121166511898 -0.42627651903944 -0.00814641175488

C 6.70605081157749 0.85419841978389 -0.13943031232844

H 6.22982590897648 2.96664908403153 -0.33393831820257

H 6.87148454159665 -1.28765403156126 0.06202436919335

H 7.78573438047245 1.01941667866377 -0.17457140089261

N 2.55121445603474 0.31758943807615 -0.01217112539715

Cartesian coordinates of molecule **5** in excited state

O -6.50485539171930 0.05663468537419 -0.11132305664808

C -2.84610629070621 0.12230747968269 0.00460038501468

C -4.16537512715227 -0.39937333457746 -0.08098345360520

C -2.64042949503782 1.55087396527746 0.11747614179099

C -1.72366203501640 -0.76258033823864 -0.02759943665042

C -5.33330307691595 0.48988063971700 -0.04581332043900

C -3.77127237002270 2.41457168554638 0.14446954949837

C -4.36080658505896 -1.79675881769695 -0.20334808886658

C -1.31574920705589 2.06080937512676 0.19880167634172

C -1.96165920612628 -2.15718868372802 -0.17689559033962

C -0.40112928269996 -0.19496868502540 0.08853401879933

C -5.05250058081971 1.91679678002184 0.06821725161695

C -3.26665307924903 -2.65331458502891 -0.25510937557367

C -0.22660076543374 1.21845630460218 0.18965490571844

H -3.59988260699751 3.49133512643536 0.22836370327224

H -5.38345827491124 -2.17118763410292 -0.26343027489875

H -1.16833333521384 3.13994706328626 0.28248333042774

H -1.13920029053103 -2.87009374956893 -0.25417198332023

H -5.91840751000352 2.58459683129631 0.08964422784618

H -3.42002066498022 -3.73035878326778 -0.36498872816106

H 0.78545842720605 1.60522695686869 0.26532189916112

N 0.66075171533195 -1.06137656186833 0.13234463429223

H 0.40048426736864 -2.04048549841983 0.21494321637664

C 2.03051509148785 -0.87810047992681 0.10267141949340

C 2.83012297881799 -2.05990573137208 0.25114391728048

C 3.89254306100998 0.46992010891262 -0.08535473701445

C 4.19317073909310 -1.93552043727972 0.23240785578745

H 2.34721037502245 -3.03132658439815 0.37856651008092

C 4.45066165103974 1.76879380789619 -0.26360768314382

C 4.78421976521716 -0.64926135500330 0.06408048486622

H 4.83515882431559 -2.81284734029718 0.34440225832471

C 5.81944566719573 1.94396487665086 -0.28763802924059

H 3.76630816886614 2.61212960025174 -0.37869262737682

C 6.18234809053632 -0.43674952967825 0.03409750377606

C 6.69439376406957 0.83787646344890 -0.13783756967693

H 6.23719631872131 2.94487469779758 -0.42355869775424

H 6.84904660888604 -1.29561757222245 0.14873792428377

H 7.77526706469200 0.99656461013927 -0.15948228986432

N 2.54643459677400 0.33002164336882 -0.06490787147588

Cartesian coordinates of molecule **6** in ground state

O -5.82063364251294 0.23059017734513 -0.07190796150717

C -2.16864032045522 -0.08008155952055 0.05215393265513

C -3.45333566128432 0.53075194504418 -0.05262783739801

C -2.06494174297623 -1.49498341431012 0.20941038174776

C -0.99268776828469 0.73312833869380 -0.01109690711062

C -4.69784795272543 -0.28855590036054 0.01304893378168

C -3.26594153334140 -2.27880081735687 0.26713700923509

C -3.55350291152097 1.90971225537735 -0.22566846513186

C -0.78953640748616 -2.06498663076158 0.30245129247020

C -1.14558827692254 2.12300913416343 -0.21269873082492

C 0.31168255529923 0.10100295305104 0.11419980322507

C -4.50999467749017 -1.72278390417856 0.17813357558648

C -2.40349398834932 2.70511900028642 -0.31295894505334

C 0.37224342995160 -1.29721290257202 0.25436581202789

H -3.15943182912263 -3.36173782548756 0.38788212960151

H -4.54981863255812 2.35029213652547 -0.30225469210157

H -0.70205086604529 -3.14756616960390 0.43359917361937

H -0.27089462140775 2.76917436685627 -0.31011281492973

H -5.41345686289821 -2.33599607459178 0.22629813920017

H -2.49131475134397 3.78280704040381 -0.46899644949653

H 1.33945743399345 -1.78605444912348 0.35969765329370

N 1.43091268905174 0.87960503909588 0.11080952902198

H 1.30094123526323 1.87032388625210 0.28176107096448

C 2.77887935581275 0.46009948635420 0.03674562007222

C 3.73490043176245 1.06824091295086 0.86589010461373

C 3.21380955690400 -0.50297083073501 -0.88926372284487

C 5.08077649223036 0.72060566666134 0.78434241646901

H 3.41477930111752 1.82885062743818 1.58343435557333

C 4.55552524743664 -0.86692591875840 -0.96048004800221

H 2.49891507506635 -0.96363886406140 -1.57380352789035

C 5.52210415077537 -0.26711645827329 -0.12308493954274

H 5.80751364688402 1.21122107087053 1.43776167366565

H 4.87210250645391 -1.61968103819681 -1.68784128107327

N 6.84424224082112 -0.66634700560420 -0.15951413752009

H 7.52594289468621 -0.01196592210508 0.21157642178742

H 7.15671176031539 -1.12091581456889 -1.01188784338459

Cartesian coordinates of molecule **6** in excited state

O -5.09301680243825 -1.58306156107047 0.92385885380627

C -2.02842601792769 0.13377607159748 -0.16256792276946

C -2.94039940926356 -0.92826326426384 0.11578677446598

C -2.40015559944522 1.49909230690220 0.08838671161256

C -0.72629221707060 -0.17132760124170 -0.69388776485556

C -4.27874393966794 -0.65480122208971 0.66741852049750

C -3.69887360336855 1.75461700257073 0.61832321045130

C -2.55200059851689 -2.26198931941913 -0.14011857844690

C -1.47264167671356 2.53365683762057 -0.19209374465934

C -0.38009497052385 -1.52614294880784 -0.93448125705134

C 0.16424754223750 0.91527479700974 -0.94439332334468

C -4.58777296812216 0.73642447677056 0.89344826719301

C -1.29117026181263 -2.54936881257264 -0.65844242827179

C -0.21093953628340 2.23998395385382 -0.69843750128549

H -3.98197771316399 2.79477076963739 0.80813685981916

H -3.27029669038040 -3.05304005468302 0.08151402622965

H -1.75688305519698 3.57172630314241 -0.00115919444557

H 0.60192306986992 -1.76569800958115 -1.34470761354880

H -5.57880814655180 0.95600729019064 1.30237677124925

H -1.00624552671000 -3.58762892279366 -0.85316063870763

H 0.50491797303080 3.03956825421953 -0.90386931421030

N 1.47443943997612 0.63781969319410 -1.44556767313658

H 1.61928230411953 0.64097744211152 -2.45475091596467

C 2.53247177695420 0.31458116302487 -0.67806855248747

C 3.79194358950111 0.01843216417071 -1.28931314080037

C 2.42979820359438 0.25242231936018 0.74619184812816

C 4.88005115107998 -0.31527601153575 -0.52753859487125

H 3.87297898137991 0.06274264998872 -2.37745650585601

C 3.51996083949244 -0.08205040118329 1.50832276456966

H 1.47490718287186 0.47546713252873 1.22049122751864

C 4.77883937700464 -0.37595639323840 0.89859608486162

H 5.83848444260317 -0.53978987670364 -0.99981442157505

H 3.43787483529083 -0.12824072822378 2.59607084549100

N 5.84174361095350 -0.70123593395707 1.64471294927681

H 6.73796237299914 -0.91088068857516 1.21817670622073

H 5.77958404019845 -0.74544587795364 2.65635166489699

Cartesian coordinates of molecule **7** in ground state

O -6.51317910049030 0.10078835220892 -0.07748499768885

C -2.85352929471369 -0.09042023132916 0.06193779842006

C -4.15635231185311 0.47355100138879 -0.07620020872033

C -2.70668921651727 -1.49038569513642 0.29878053036317

C -1.70303642783203 0.75397439165310 -0.04729613098741

C -5.37508444657365 -0.37860491225094 0.03534906649251

C -3.88281179024252 -2.30630278030104 0.40007922945511

C -4.29925480317607 1.83708031656091 -0.32512809154848

C -1.41434662378155 -2.01437696948058 0.42546427800480

C -1.89905175241514 2.12535787075640 -0.32375590814567

C -0.37959141895284 0.16982095233867 0.11152774346349

C -5.14346511296712 -1.79463308812901 0.28007392174068

C -3.17443015254911 2.66182829517227 -0.45539963597885

C -0.27671609497308 -1.21568758226451 0.33329499616993

H -3.74371251544213 -3.37710289084781 0.58101248758140

H -5.30886545189007 2.24103681535424 -0.42609259366621

H -1.29414545153302 -3.08442155778619 0.61881701918585

H -1.04540992831495 2.79328843446661 -0.45533276917225

H -6.02756973664112 -2.43196197104135 0.36236220256398

H -3.29517163321234 3.72633052875965 -0.66929549008792

H 0.70443509939579 -1.66817133492545 0.46710858818445

N 0.71467483853865 0.98061683138273 0.06046058784979

H 0.55185403159735 1.97560867051528 0.16422090127201

C 2.07624769842220 0.60541793646394 0.01358803097600

C 3.01192859158580 1.31398669380652 0.78275086915575

C 2.55023976729126 -0.41313401543097 -0.82857762211118

C 4.36971025053318 1.01393020998555 0.72978064362978

H 2.66619784482051 2.12003949300258 1.43616195374136

C 3.90317301206762 -0.73473152352758 -0.87653467349793

H 1.85532254968697 -0.95762604000978 -1.47070936255709

C 4.85850148526008 -0.03427548061098 -0.09349162527587

H 5.05398250462877 1.59802464752169 1.34384861141286

H 4.21921230605514 -1.53348966303343 -1.54628542298725

N 6.19613586138386 -0.35264124252808 -0.13617365901410

C 6.66447112603904 -1.40107936218986 -1.02153488933438

H 6.19388685863036 -2.37457750731704 -0.79258639204299

H 6.45732967821647 -1.17502944508274 -2.08451957485770

H 7.74923470270777 -1.51565332558211 -0.90468125793760

C 7.14850312644252 0.40845634852307 0.64871378515534

H 7.16760091884204 1.47694767063297 0.36230893947789

H 6.92398482588681 0.35967435753132 1.72980385722583

H 8.15427049553889 -0.00179484622013 0.49476452369001

Cartesian coordinates of molecule **7** in excited state

O -5.36346615802243 0.10235310680729 -2.16380406878267

C -2.48913846464588 -0.27198706675092 0.09862736882553

C -3.35404754863144 0.29793870899174 -0.88344695629270

C -2.80766253122209 -1.53491843417437 0.70568225911049

C -1.28920180149447 0.42614420807015 0.47794640305665

C -4.59002512020431 -0.38877205584469 -1.29717312872039

C -4.00614496907164 -2.19186213657743 0.29961883727916

C -3.01817233899636 1.53944570173722 -1.46743750946112

C -1.92889222967549 -2.08064910981036 1.67449047194939

C -0.99308849560442 1.67075076223411 -0.13573573260858

C -0.44356195541765 -0.17948952181852 1.45528454179522

C -4.85013191552468 -1.65056044926705 -0.64762986624939

C -1.85536327427030 2.21024227184173 -1.09432856362906

C -0.76645890787280 -1.40926258380285 2.03843182621259

H -4.24865564930888 -3.15361682888161 0.76243754700272

H -3.69824366261853 1.95097802941530 -2.21508476875924

H -2.17282385093791 -3.04188996930752 2.13413408593777

H -0.08884779187489 2.20993788342229 0.15021137426838

H -5.76467908714254 -2.17343545662021 -0.94386232168943

H -1.61001696012639 3.17204815817838 -1.55455927492580

H -0.08744808904097 -1.83583921918073 2.78067562104380

N 0.76243759633747 0.48372699359021 1.84146336552322

H 0.73761975519493 1.10213908183826 2.65127670590360

C 1.93470387529648 0.38175671693212 1.18516285591339

C 3.08038014620823 1.09728730138539 1.64947683962248

C 2.07256231991615 -0.42885190437841 0.01952173776988

C 4.28386120740811 1.00421673308215 1.00002456761996

H 2.98539879975705 1.72471704791878 2.53836035896080

C 3.27606521522784 -0.51990540878001 -0.63452995219933

H 1.20911930213101 -0.98130892105700 -0.34933087629318

C 4.43113490253302 0.18926556102408 -0.17083081958676

H 5.13177702020032 1.56500516717206 1.38780117432423

H 3.34068476536550 -1.15089417876474 -1.51858045365836

N 5.61766388204690 0.09362650622699 -0.81241756899055

C 5.74907242207506 -0.71859538114747 -2.01956797558507

H 5.56447452905111 -1.78219354626036 -1.80089835722702

H 5.03845270425197 -0.39004018882226 -2.79280866883397

H 6.76558755924031 -0.61205655317310 -2.41179530415213

C 6.79784843580545 0.79173928478797 -0.30686400233785

H 6.64763373571790 1.88243738071890 -0.32432439980747

H 7.02430018805713 0.48348155729790 0.72506442099586

H 7.65568507488213 0.54595561804656 -0.94101146702504

Cartesian coordinates of molecule **8** in ground state

O -5.43865288624094 0.45487321079756 -0.00136709123587

C -1.81384930459906 -0.08205641119174 -0.00104225409683

C -3.05913310840023 0.61250733719959 0.00201088404089

C -1.80033028359589 -1.50990806758938 -0.00525244514571

C -0.58773138167822 0.65646544702483 0.00179649628607

C -4.35119664332651 -0.13388533880101 -0.00245185852823

C -3.05129651206284 -2.22584970283313 -0.00804242098537

C -3.07925931301361 2.00340970644401 0.01029088317149

C -0.57105803506604 -2.16604115604825 -0.00535412425154

C -0.66198526375010 2.07079228083428 0.01444874614328

C 0.66865806987731 -0.07349520611046 -0.00599085718710

C -4.25496624234935 -1.59057196942934 -0.00724424726666

C -1.88160171283733 2.73247578266858 0.01799279253525

C 0.64442503088509 -1.47161903045898 -0.00565536401246

H -3.00714286119051 -3.31944851131066 -0.01102292339528

H -4.04709343329507 2.50907579347322 0.01239465926441

H -0.55171202811651 -3.25955404017882 -0.00714995705485

H 0.23915770580486 2.68757237246810 0.02540277248926

H -5.19633711187100 -2.14553285682625 -0.00983476420109

H -1.90378485647287 3.82450078190897 0.02804241704458

H 1.58538320527398 -2.01239703534738 -0.00709233141314

N 1.83833950265039 0.66041021633729 -0.01651632530512

H 1.72073871814708 1.66573234224454 -0.04161681885398

C 3.16693226726453 0.28101320967133 -0.00542810338234

N 3.53222980184047 -1.00076956100980 0.02903825930270

C 4.14807803941804 1.31142695465840 -0.03095073989162

C 4.84464923019746 -1.26369684150287 0.03819601291360

N 5.44033656596859 1.03922846182687 -0.02169865716951

H 3.83818028991468 2.36357656284281 -0.05885041915351

C 5.80342836611251 -0.25534896389708 0.01300218191041

H 5.14199477867568 -2.31750574207921 0.06656833244084

H 6.87221627013542 -0.48996695978604 0.02069254358746

Cartesian coordinates of molecule **8** in excited state

O -5.43364173727636 0.47122122019825 -0.00555553939802

C -1.81542478367714 -0.07463450434714 -0.00231166183313

C -3.05550631013615 0.61748140497428 -0.00318769405197

C -1.79776114074800 -1.52427733319395 -0.00260344585358

C -0.58722938161523 0.65699384271295 -0.00104792010169

C -4.32799909144608 -0.11296579904845 -0.00456826743228

C -3.02924117484983 -2.23403753884118 -0.00410899241526

C -3.07019311004638 2.03473513143914 -0.00271088953672

C -0.54971881134835 -2.20867770375819 -0.00147789806655

C -0.64405846542188 2.07944730948210 -0.00046948863609

C 0.65007562054690 -0.08728162478526 -0.00022980001929

C -4.23598630646909 -1.56923784816779 -0.00512069852686

C -1.87473301555514 2.74391178764807 -0.00136031068769

C 0.63909142709117 -1.51769090596707 -0.00036123545342

H -2.99897419878909 -3.32705544042028 -0.00446735972426

H -4.03668060056942 2.54018557592987 -0.00331998621579

H -0.54549800282006 -3.30090052791521 -0.00172785737962

H 0.25955050980497 2.69209807156673 0.00086578681342

H -5.18164965034893 -2.11850538197857 -0.00628303971510

H -1.88706512085570 3.83724122544831 -0.00083638740923

H 1.59227302167470 -2.03847380282981 0.00031791662867

N 1.81905275232184 0.62552492892347 -0.00008520621728

H 1.69385374414446 1.63386950642732 -0.00311879084886

C 3.15111310044351 0.26454775733801 0.00331657914544

N 3.53234612150160 -1.01868861500694 0.00988525707087

C 4.11257002865619 1.31266606785855 -0.00012412699107

C 4.84295642970665 -1.25842569204485 0.01324465919188

N 5.41011827085918 1.06095285526876 0.00340084005274

H 3.78499957345796 2.35981233567632 -0.00586124386082

C 5.78913908467317 -0.22837393600751 0.01021317290160

H 5.16173161099968 -2.30623806064272 0.01864746940432

H 6.86074688649084 -0.44976438003723 0.01334844006565

**References**

1. Sandoval-Altamirano C, De la Fuente JR, Berrios E, et al (2018) Photophysical characterization of hydroxy and ethoxy phenalenone derivatives. J Photochem Photobiol A Chem 353:349–357. https://doi.org/10.1016/j.jphotochem.2017.11.049

2. Sandoval-Altamirano C, Berrios E, Morales J, et al (2023) Phenalenone Derivatives: The voyage from Photosensitizers to Push-Pull fluorescent molecules. J Photochem Photobiol A Chem 114587. https://doi.org/10.1016/j.jphotochem.2023.114587

3. Kaye EG, Kailass K, Sadovski O, Beharry AA (2021) A Green-Absorbing, Red-Fluorescent Phenalenone-Based Photosensitizer as a Theranostic Agent for Photodynamic Therapy. ACS Med Chem Lett 12:1295–1301. https://doi.org/10.1021/acsmedchemlett.1c00284
